# Emergence of Near-Infrared Photoluminescence via ZnS Shell Growth on the AgBiS_2_ Nanocrystals

**DOI:** 10.1021/acs.chemmater.4c02406

**Published:** 2024-12-11

**Authors:** Asim Onal, Tarik Safa Kaya, Önder Metin, Sedat Nizamoglu

**Affiliations:** † Graduate School of Biomedical Science and Engineering, Koç University, Istanbul 34450, Türkiye; ‡ Graduate School of Material Science and Engineering, Koç University, Istanbul 34450, Türkiye; § Department of Chemistry, College of Sciences, Koç University, Istanbul 34450, Türkiye; ∥ Department of Electrical and Electronics Engineering, Koç University, Istanbul 34450, Türkiye

## Abstract

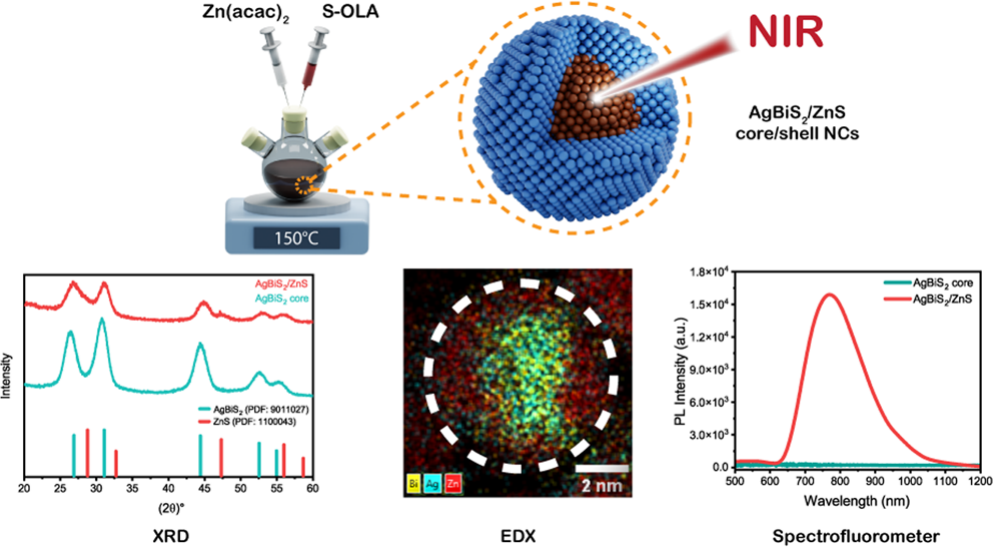

AgBiS_2_ nanocrystals (NCs), composed of nontoxic, earth-abundant materials and exhibiting an exceptionally high absorption coefficient from visible to near-infrared (>10^5^ cm^–1^), hold promise for photovoltaics but have lack of photoluminescence (PL) due to intrinsic nonradiative recombination and challenging shell growth. In this study, we reported a facile wet-chemical approach for the epitaxial growth of ZnS shell on AgBiS_2_ NCs, which triggered the observation of PL emission in the near-infrared (764 nm). Since high quality of the core is critical for epitaxial shell growth, we first obtained rock-salt structured AgBiS_2_ NCs with high crystallinity, nearly spherical shape and monodisperse size distribution (<6%) via a dual-ligand approach reacting Ag–Bi oleate with elemental sulfur in oleylamine. Next, a zincblende ZnS shell with a low-lattice mismatch of 4.9% was grown on as-prepared AgBiS_2_ NCs via a highly reactive zinc (Zn(acac)_2_) precursor that led to a higher photoluminescence quantum yield (PLQY) of 15.3%, in comparison with a relatively low reactivity precursor (Zn(ac)_2_) resulting in reduced PLQY. The emission from AgBiS_2_ NCs with ultrastrong absorption, facilitated by shell growth, can open up new possibilities in lighting, display, and bioimaging.

## Introduction

Colloidal quantum dots (QDs) have been extensively utilized in various applications, including solar cells, display technology, optical sensing, and biological labeling.^1−4^ Historically, QDs made from II–VI and IV–VI binary semiconductors, such as cadmium-based (CdE, E: Se, S, Te) and lead-based (PbE, E: Se, S, Te) compounds, have been prominent due to their exceptional optical characteristics.^5−10^ However, the inherent toxicity of these materials has prompted a shift in research toward developing QDs that are free from cadmium and lead, offering a safer and more environmentally sustainable alternative.

AgBiS_2_ nanocrystals (NCs) are emerging promising candidates for their composition of nontoxic, earth-abundant elements, broad absorption spectrum spanning from the visible to near-infrared region, high air stability, and favorable solution processability.^11−14^ A particularly important feature of AgBiS_2_ NCs is their high absorption coefficient (∼10^5^ cm^–1^), which is substantially higher than the other NCs such as CdTe,^15^ GaAs,^16^ InP,^17,18^ and perovskites,^19^ over a broad spectral range (400–1000 nm).^13^ This high absorption coefficient allows for thin and flexible photoactive layers while maintaining highly effective functionality. These superior properties have led to the utilization of AgBiS_2_ NCs in various applications, including antibacterial treatments, solar energy harvesting, and neuron photostimulation.^11,20−22^ This property not only reduces the amount of material needed but also lowers costs, enhancing both environmental sustainability and biocompatibility.

The core/shell nanostructures, where the core NCs are coated with shell materials, is an effective method to reduce the density of surface defects,^23,24^ extend photoluminescence (PL) lifetime,^25^ and enhance chemical,^26^ thermal,^27^ and physical stability compared to core NCs.^28^ Although compositional variations of AgBiS_2_ have been extensively studied to date,^29−32^ its core/shell nanostructure has not been reported yet. Notably, ZnS is commonly used as a shell material because it has a wide bandgap, which effectively passivates the surface states of many core materials, thereby improving their optical properties and stability. Moreover, it also has a low lattice mismatch with AgBiS_2_ that can allow for the efficient shell growth.^33,34^ However, typical ZnS shelling techniques, such as those employed for coating CdSe,^35,36^ Ag_2_S,^37^ Ag_2_ZnSnS_4_,^38,39^ and CuInS_2_^40^ QDs, are ineffective for AgBiS_2_. Second, the high temperatures used for ZnS shelling^41,42^ can adversely affect the AgBiS_2_ core. The high temperatures can cause degradation or alteration of the AgBiS_2_ crystal structure and surface properties, thereby hindering the ZnS shell growth.

In this study, we report the synthesis of AgBiS_2_/ZnS core/shell NCs, leading to the observation of photoluminescence by AgBiS_2_ for the first time. For that, we initially optimized the crystallinity, shape and uniformity of the AgBiS_2_ NCs. Following this, ZnS shell was grown on the AgBiS_2_ NCs by decorating the NCs surface with oleylamine (OLA) and oleic acid (OA) ligands. The formation of AgBiS_2_/ZnS core/shell NCs was verified by the X-ray diffraction (XRD), X-ray photoelectron spectroscopy (XPS), transmission electron microscopy (TEM), and high-angle annular dark-field scanning TEM (HAADF-STEM) analysis. The as-synthesized AgBiS_2_/ZnS NCs exhibit photoluminescence emission at 764 nm with the photoluminescence quantum yield (PLQY) of 15.3%. Moreover, we investigated the influence of different parameters, including the type of zinc and sulfur precursors, the reaction time, and temperature on the optical properties of AgBiS_2_/ZnS NCs.

## Results

### AgBiS_2_ Core NC Synthesis

**Figure 1:**
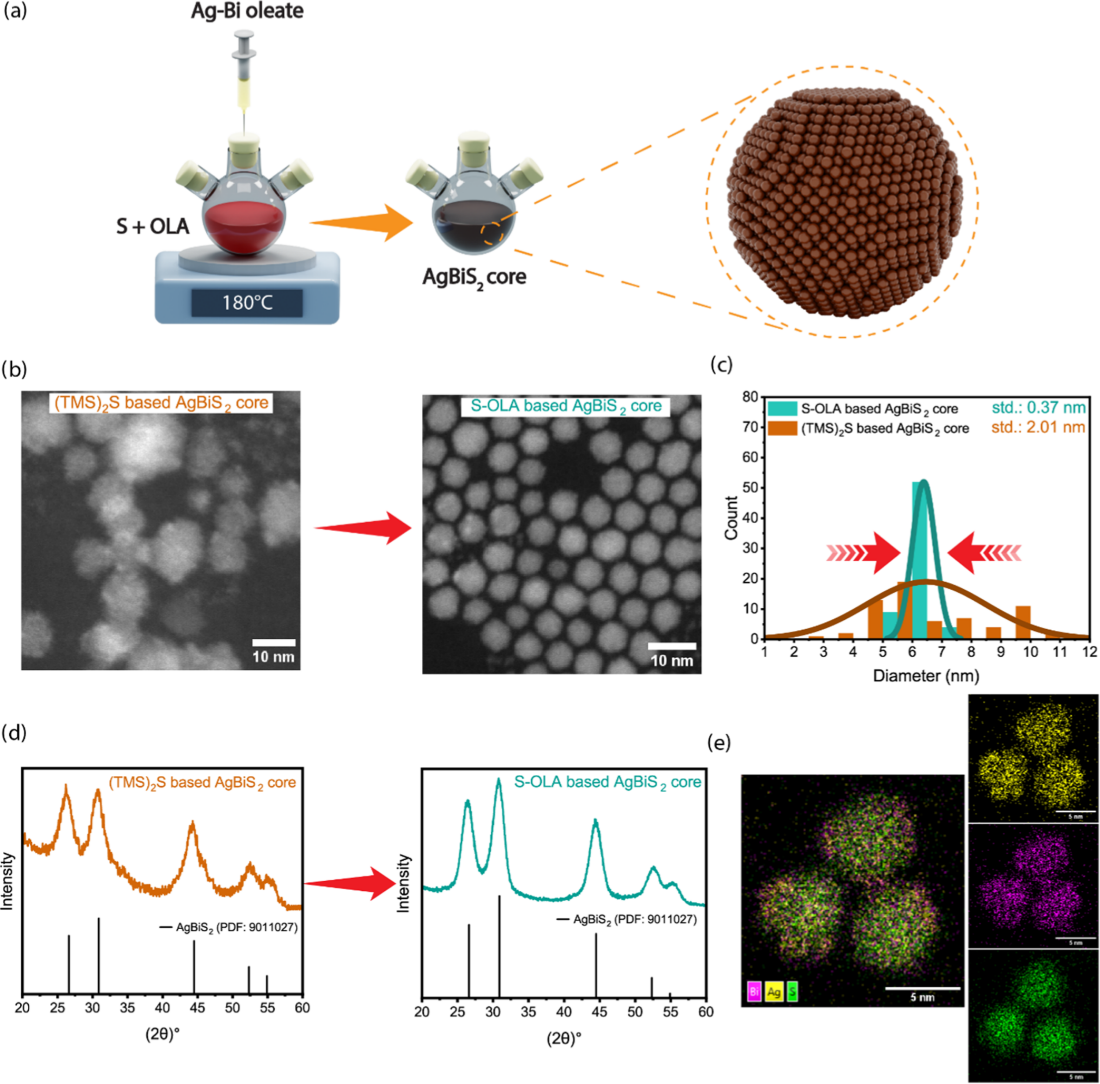
(a) Schematic illustration of the synthetic procedure used for the AgBiS_2_ core NCs. (b) Dark-field transmission electron microscopy (TEM) images of (TMS)_2_S-based AgBiS_2_ core NCs and S-OLA-based AgBiS_2_ core NCs. (TMS)_2_S-based AgBiS_2_ core NCs led to irregular NC shapes and sizes, whereas S-OLA-based NCs improved the NC morphology toward more spherical shapes with similar sizes of NCs. (c) Size distribution of the (TMS)_2_S-based AgBiS_2_ core NCs and S-OLA-based AgBiS_2_ core NCs clearly indicates that NCs sizes narrowed from 31 to 6% when the sulfur source is selected as S-OLA. (d) X-ray diffraction (XRD) patterns of (TMS)_2_S-based AgBiS_2_ core NCs and S-OLA based AgBiS_2_ core NCs. The S-OLA based AgBiS_2_ NCs exhibit narrower diffraction peaks, indicating a higher degree of crystallinity compared to the (TMS)_2_S-based NCs (PDF card number for cubic AgBiS_2_: 9011027). (e) HAADF-STEM image of S-OLA based AgBiS_2_ core NCs. The EDX maps show the distributions of Ag, Bi, and S along with a superimposed Ag–Bi–S element map.

The synthesis of AgBiS_2_ NCs was adapted based on the procedure reported by Li et al.,^43^ which utilizes oleate precursors of silver acetate (Ag(OAc)) and bismuth acetate (Bi(OAc)_3_) in a reaction with elemental sulfur and oleylamine (abbreviated as S-OLA hereafter) (Figure 1a) (The details of our modified synthesis are provided in the Supporting Information). Unlike the conventional methods using bis(trimethylsilyl) sulfide (TMS)_2_S,^11,13,44−46^ which necessitates a lower reaction temperature of 100 °C due to high reactivity of (TMS)_2_S, the use of S-OLA allowed to the synthesis at a higher temperature of 180 °C. Therefore, this approach significantly improved the crystallinity, uniformity, and monodispersity of AgBiS_2_ NCs, which is crucial for effective subsequent ZnS shell coating (Figure 1b–d).

TEM analysis revealed that AgBiS_2_ NCs synthesized by using S-OLA exhibited nearly spherical-shaped monodisperse NCs with an average diameter of 6.38 ± 0.37 nm (Figure 1b, right). In contrast, AgBiS_2_ NCs synthesized by using TMS_2_S as the sulfur source showed nonuniform shapes with an average diameter of 6.47 ± 2.01 nm (Figure 1b, left). Moreover, the size distribution analysis demonstrated a significant decrease in the standard deviation from 31 to 6% with S-OLA (Figure 1c), indicating a transition from poly to monodispersity. XRD analysis further confirmed the enhanced crystallinity of AgBiS_2_ NCs synthesized with S-OLA, as indicated by narrower full width at half-maximum (fwhm) of the diffraction peaks (Figure 1d). High-angle annular dark-field scanning transmission electron microscopy (HAADF-STEM) and energy-dispersive X-ray spectroscopy (EDX) demonstrated uniform elemental distributions within the NCs (Figure 1e), highlighting S-OLA’s effectiveness as a sulfur precursor for the controlled synthesis and superior quality of AgBiS_2_ NCs.

### ZnS Shell Growth

**Figure 2:**
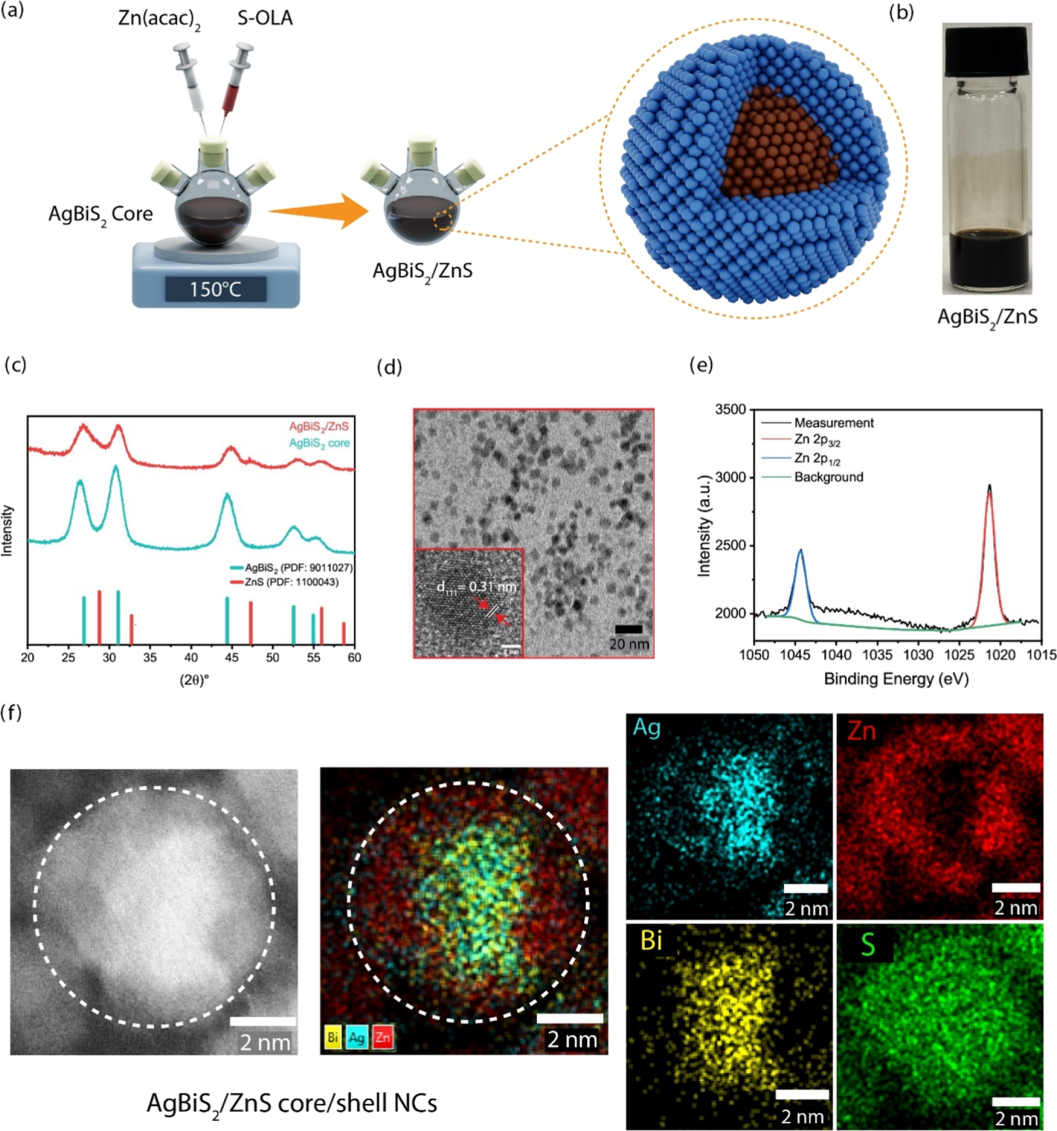
(a) Schematic illustration of the synthetic procedure used for the ZnS shell growth on AgBiS_2_ core NCs. (b) Photograph of AgBiS_2_/ZnS NCs under ambient light. (c) XRD patterns of AgBiS_2_ core NCs and AgBiS_2_/ZnS core/shell NCs, with reference patterns for cubic AgBiS_2_ (PDF card no: 9011027) and cubic ZnS (PDF card no: 1100043). (d) Transmission electron microscopy (TEM) and high-resolution TEM (HRTEM) images (inset) of AgBiS_2_/ZnS core/shell NCs. (e) X-ray photoelectron spectroscopy (XPS) analysis of the Zn 2p spectrum in the as-synthesized AgBiS_2_/ZnS core/shell NCs. (f) HAADF-STEM image of AgBiS_2_/ZnS core/shell NCs and corresponding EDX data showing the distributions of Ag, Bi, Zn, and S along with a superimposed Ag–Bi–Zn element map.

For the ZnS shell growth, we developed a new synthetic approach comprising the use of dual ligands OA and OLA, which is helpful for preventing the aggregation and limiting oxidation of the NCs during the reaction.^47^ The ZnS shell growth is achieved through dropwise hot-injection of zinc acetylacetonate (Zn(acac)_2_) and S-OLA as zinc and sulfur precursors (*T* = 150 °C), respectively (Figures 2a and S1). The inclusion of OLA as a cosurfactants enhances the shell quality compared to OA alone,^47^ enabling the growth of a zinc blende crystalline structure ZnS on the AgBiS_2_ core NCs (Figure 2b). To minimize the probability of Ostwald ripening during the shell growth, a lower growth temperature was chosen compared to the one used in synthesis of AgBiS_2_ core NCs.^48^

The crystal structure of the synthesized AgBiS_2_ core and AgBiS_2_/ZnS core/shell NCs was investigated by using XRD analysis. The XRD patterns (Figure 2c) reveal the presence of well-defined diffraction peaks for both samples. The AgBiS_2_ core exhibits characteristic peaks at 26.56, 31.05, 45.03, 53.35, and 56.22°, corresponding to the (111), (200), (220), (311), and (222) facets of the rock-salt AgBiS_2_ crystal structure (PDF card no. 9011027).^33^ Notably, the XRD pattern of the AgBiS_2_/ZnS core/shell NCs displays a slight shift in these peaks toward higher diffraction angles toward the standard positions of zinc blende bulk crystal structure of ZnS (PDF card no. 1100043)^34^ compared to the pristine AgBiS_2_ core NCs. This slight shift suggests a minor lattice parameter modification upon ZnS shell formation on the AgBiS_2_ cores.^49^ Based on the lattice constants (5.69 Å for rock-salt AgBiS_2_, and 5.41 Å for zinc blende ZnS), the calculated low-lattice mismatch of 4.9% suggests a suitable condition for effective shell growth.

Figure 2d presents representative bright-field TEM and high-resolution TEM (HRTEM) images of the corresponding AgBiS_2_/ZnS core/shell NCs. The TEM image reveals that there is no significant change in the morphology of AgBiS_2_ after the ZnS shell formation with a nearly spherical shape. HRTEM analysis further confirms the successful core/shell structure formation and the high crystallinity of the NCs (Figure 2d, inset). The HRTEM image reveals continuous lattice fringes throughout the entire nanoparticle, signifying a well-ordered crystalline structure. For the AgBiS_2_ core NCs, the measured interplanar spacing of these fringes corresponds to 0.32 nm, which matches well with the expected *d*-spacing of the (111) lattice plane in the AgBiS_2_ crystal structure (Figure S2) (PDF card no. 9011027).^33^ In the case of the ZnS shell, the measured interplanar spacing is 0.31 nm, which aligns with the expected *d*-spacing of the (111) lattice plane in the zinc blende crystal structure of ZnS (PDF No. 1100043).^34^ These observations provide compelling evidence for the successful growth of a crystalline ZnS shell onto the AgBiS_2_ cores.

To further substantiate the formation of the ZnS shell, we conducted X-ray photoelectron spectroscopy (XPS) analysis on both AgBiS_2_ core and AgBiS_2_/ZnS core/shell NCs. For AgBiS_2_ NCs, two prominent peaks at 367.1 and 373.2 eV (Figure S3a), with a spin–orbit splitting of 6.1 eV, correspond to Ag 3d_5/2_ and Ag 3d_3/2_, respectively, confirming the presence of Ag(I) states.^14,50^ Peaks at 157.7 and 163.0 eV are attributed to Bi 4f_7/2_ and Bi 4f_5/2_ (Figure S3b). The separation between the spin–orbit split components of the Bi 4f peaks was measured to be 5.3 eV. This energy difference is indicative of the presence of Bi(III) species.^51^ The peaks at 160.6 and 161.7 eV are assigned to S 2p_3/2_ and S 2p_1/2_, respectively (Figure S3b).^14^ Likewise, the energy difference between the spin–orbit split component of the S 2p peaks was found to be 1.1 eV that confirms the presence of sulfur in the −2 oxidation state.^52^ The Zn core-level spectrum shown in Figure 2e displays a split into Zn 2p_1/2_ at 1044.4 eV and Zn 2p_3/2_ at 1021.4 eV, with a spin–orbit splitting energy of 23.0 eV. This observation indicates that the Zn is in the +2 oxidation state.^53^ The presence of these peaks provides definitive evidence of Zn incorporation, confirming the successful formation of the ZnS shell. The distinct Zn peaks are not observed in the spectra of the AgBiS_2_ cores alone, underscoring that the Zn signals originate exclusively from the ZnS shell. This substantial presence of Zn in the core/shell structure highlights the successful integration of ZnS onto the AgBiS_2_ core NCs.

Table S1 illustrates the resulting atomic percentages of Ag, Bi, S, and Zn in the studied samples, including TMS-based AgBiS_2_, S-OLA-based AgBiS_2_, and AgBiS_2_/ZnS core/shell NCs with varying Zn/S molar ratios (0.3, 0.6, 0.9, and 1.2). A trend emerges where higher Zn/S molar ratios, which correspond to increased Zn incorporation and reduced Ag and Bi atomic percentages, lead to a progressive blue shift in the PL emission peaks (Figure 4e and Table S1). This stoichiometric-sensitive behavior may originate from possible diffusion of Zn^2+^ ions into the core and alteration of the core/shell interface.

HAADF-STEM analysis was further employed to investigate the elemental distribution within the AgBiS_2_/ZnS core/shell NCs (Figure 2f). The HAADF-STEM image provides complementary information to the XRD data, offering insights into the spatial arrangement of elements at the nanoscale. The corresponding energy-dispersive X-ray spectroscopy (EDX) data corroborates the presence of a ZnS shell surrounding the AgBiS_2_ core NCs. Elemental mapping alongside EDX analysis provides a more comprehensive picture of the elemental distribution. The mapping images reveal a uniform sulfur (S) distribution throughout the entire structure, reflecting its presence in the AgBiS_2_ core and the ZnS shell. In contrast, Ag and Bi primarily localize within the inner core region, while Zn concentrates in the outer shell. This observation strongly supports the successful formation of core/shell structures with distinct elemental compositions.

**Figure 3:**
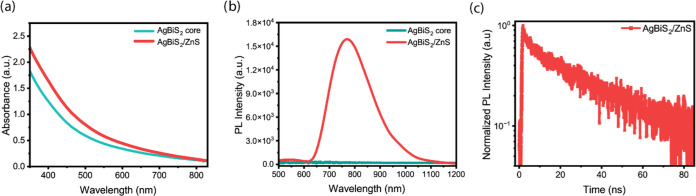
(a) UV–vis–NIR absorption spectra of AgBiS_2_ core NCs and AgBiS_2_/ZnS core/shell NCs. (b) Photoluminescence (PL) spectra of both AgBiS_2_ core NCs and AgBiS_2_/ZnS core/shell NCs (λ_exc_ = 310 nm). (c) Time-resolved photoluminescence (TRPL) decay curve of AgBiS_2_/ZnS core/shell NCs. (λ_exc_ = 375 nm).

Figure 3a presents the absorption spectra of the AgBiS_2_ core NCs and AgBiS_2_/ZnS core/shell NCs. The core/shell NCs exhibit an enhanced absorption intensity at higher energies than the AgBiS_2_ core NCs due to the ZnS shell. Therefore, the absorption spectra of the core/shell structure converge with that of the AgBiS_2_ core, reflecting the dominance of the intrinsic absorption properties of the core material at lower energies. Consistent with prior findings, pristine AgBiS_2_ core NCs exhibited no photoluminescence (PL) emission (Figure 3b). This behavior is likely attributed to the dominance of large surface trap states within the core material. Remarkably, core/shell structures displayed a clear PL peak centered at 764 nm with a FWHM of 185 nm (Figure 3b). The PLQY of the core/shell NCs is 15.3% with an average lifetime of 34.3 ns (Figure 3c). To the best of our knowledge, these values represent the first reported instance of achieving detectable PL emission from AgBiS_2_.

### Influence of Zinc and Sulfur Precursor Selection on ZnS Shell Formation

**Figure 4:**
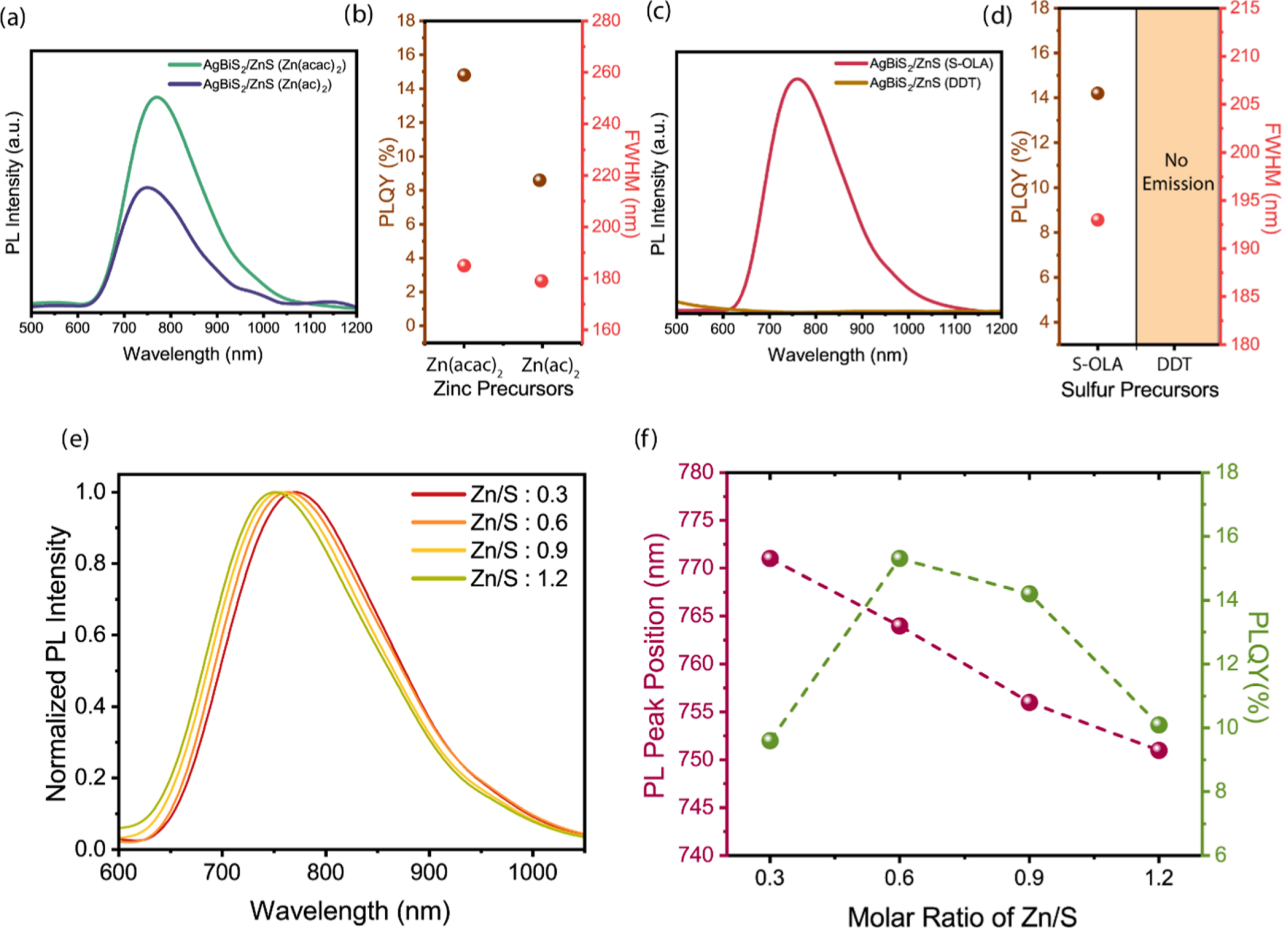
Photoluminescence (PL) spectra of AgBiS_2_/ZnS core/shell nanocrystals (NCs) (λ_exc_ = 310 nm) for different (a) zinc and (c) sulfur precursors. (b) Comparative analysis of photoluminescence quantum yield (PLQY) and full width at half-maximum (FWHM) by different zinc and sulfur precursor types. (e) Normalized PL intensity of AgBiS_2_/ZnS core/shell NCs as a function of Zn/S molar ratios from 0.3 to 1.2 (λ_exc_ = 310 nm). (f) Comparison of the PL peak positions and PLQY of AgBiS_2_/ZnS NCs for various Zn/S molar ratios.

To investigate the impact of shell precursor reactivity, various reactive precursors were tested as zinc and sulfur sources. As zinc sources, Zn(acac)_2_ (Δ_f_*H* = −949.9 kJ/mol)^54^ and Zn(ac)_2_·2H_2_O (Δ_f_*H* = −1669.3 kJ/mol)^55^ were selected as high-reactive and low-reactive Zn-precursors, respectively (Figure 4a,b). For sulfur sources elemental sulfur dissolved in OLA (S-OLA, Δ_f_*H* = 0 kJ/mol)^56^ was used as a high reactive S-precursor, whereas DDT (Δ_f_*H* = −328.1 kJ/mol)^57^ was selected as a low-reactive precursor (Figure 4c,d). Studies have indicated that at high temperatures, elemental sulfur interacts with OLA (as well as other alkylamines) to generate H_2_S along with several reactive sulfur-containing compounds (like alkylthioamides and dialkylamidines).^58^ These compounds are likely the actual reactive sulfur precursors in the reactions performed in the developed recipe. In the synthesis of various core/shell NCs, elemental sulfur dissolved in trioctylphosphine (TOP) is commonly utilized as a sulfur source.^28,59,60^ However, we deliberately chose not to use this precursor because its strong binding affinity to Ag(I) would likely facilitate cation exchange processes instead of epitaxial shell growth by encouraging the formation of Ag(I) vacancies.^61^ Our findings indicate that the low reactivity of the Zn precursor, Zn(ac)_2_, resulted in insufficient interaction with the S precursors, thereby hindering effective ZnS shell formation on the AgBiS_2_ cores. Consequently, this limited shell growth led to a lower PLQY of 8.6%, with a PL peak observed at 749 nm and a fwhm of 179 nm. Conversely, a highly reactive precursor (Zn(acac)_2_) can efficiently react with the S-precursors to form ZnS monomers rapidly, promoting epitaxial shell growth. This results in a higher PLQY of 14.8%, with a PL peak at 767 nm and an FWHM of 185 nm (Figure 4a,b). The observed blue-shift in the emission of AgBiS_2_/ZnS NCs when using the low-reactive Zn precursor (Zn(ac)_2_) also suggests that etching, cation exchange, or alloying are more probable than epitaxial shell growth.^41^

Figure 4c,d demonstrate that the effect of the different reactive S-precursors on the AgBiS_2_/ZnS core/shell NCs. The use of low-reactive S-precursor (DDT) in the shell growth reaction results in a slow conversion rate from precursor to monomer. This sluggish conversion permits the Zn-precursor to remain available for binding to the NCs surface, thereby facilitating potential cation exchange.^41^ Consequently, this results in the absence of photoluminescence emission from the AgBiS_2_/ZnS core/shell NCs. Conversely, when a highly reactive sulfur precursor like S-OLA was employed, the rapid reaction between the Zn and S precursors on the surface of the AgBiS_2_ core NCs resulted in a high PLQY of 14.2% with a PL peak at 758 nm and fwhm of 193 nm.

To further investigate the impact of core quality on shell formation, we performed a control synthesis by using the determined zinc and sulfur precursors with (TMS)_2_S (Figure 1b (left)). HAADF-STEM analysis shows that Ag and Bi are uniformly distributed throughout the interior and surface of the NCs, whereas Zn does not show surface localization (Figure S4a). Moreover, NCs do not have any detectable PL (Figure S4b,c). As another control group, we injected identical quantities of Zn and S precursors into the reaction mixture, omitting the AgBiS_2_ cores. The absence of PL in the near-infrared (NIR) spectral region in this control experiment confirms that the observed NIR emission originates specifically from the AgBiS_2_ core NCs (Figure S5a,b). These observations strongly indicate that the quality of the AgBiS_2_ core critically influences the successful formation of core/shell structure and PL emission.

### Influence of Varying Zn/S Precursor Ratios on the ZnS Shell Formation on AgBiS_2_ Core NCs

We investigated the effect of different Zn to S molar ratios on the PL properties of AgBiS_2_/ZnS core/shell NCs (Figure 4e,f). While the emission peak was at 771 nm with a PLQY of 9.6% for a Zn/S ratio of 0.3, increasing the Zn/S ratio to 0.6 shifts the emission to 764 nm and improves PLQY to 15.3%. Increasing the Zn/S ratio to 0.9 and 1.2 blue-shifts the emission peaks to 756 and 751 nm, respectively, possibly due to interdiffusion of Zn atoms into the AgBiS_2_ core.^62^ For the ratios the PLQYs also decreased to 14.2 and 10.1%, respectively, due to the formation of interfacial dislocations by lattice strain that elevates the density of defects at the interface, thereby increasing the rate of nonradiative recombination.^63,64^ The photoluminescence lifetime measurements reveal a decreasing trend of average lifetime while the Zn/S molar ratio increases (Figure S11 and Table S2). These findings highlight the critical balance required in the Zn/S ratio, while a ratio of 0.6 providing optimal conditions for maximizing PLQY and achieving effective shell growth without introducing significant defects.

### Temperature and Time Optimization for the Synthesis of AgBiS_2_/ZnS Core/Shell NCs

**Figure 5:**
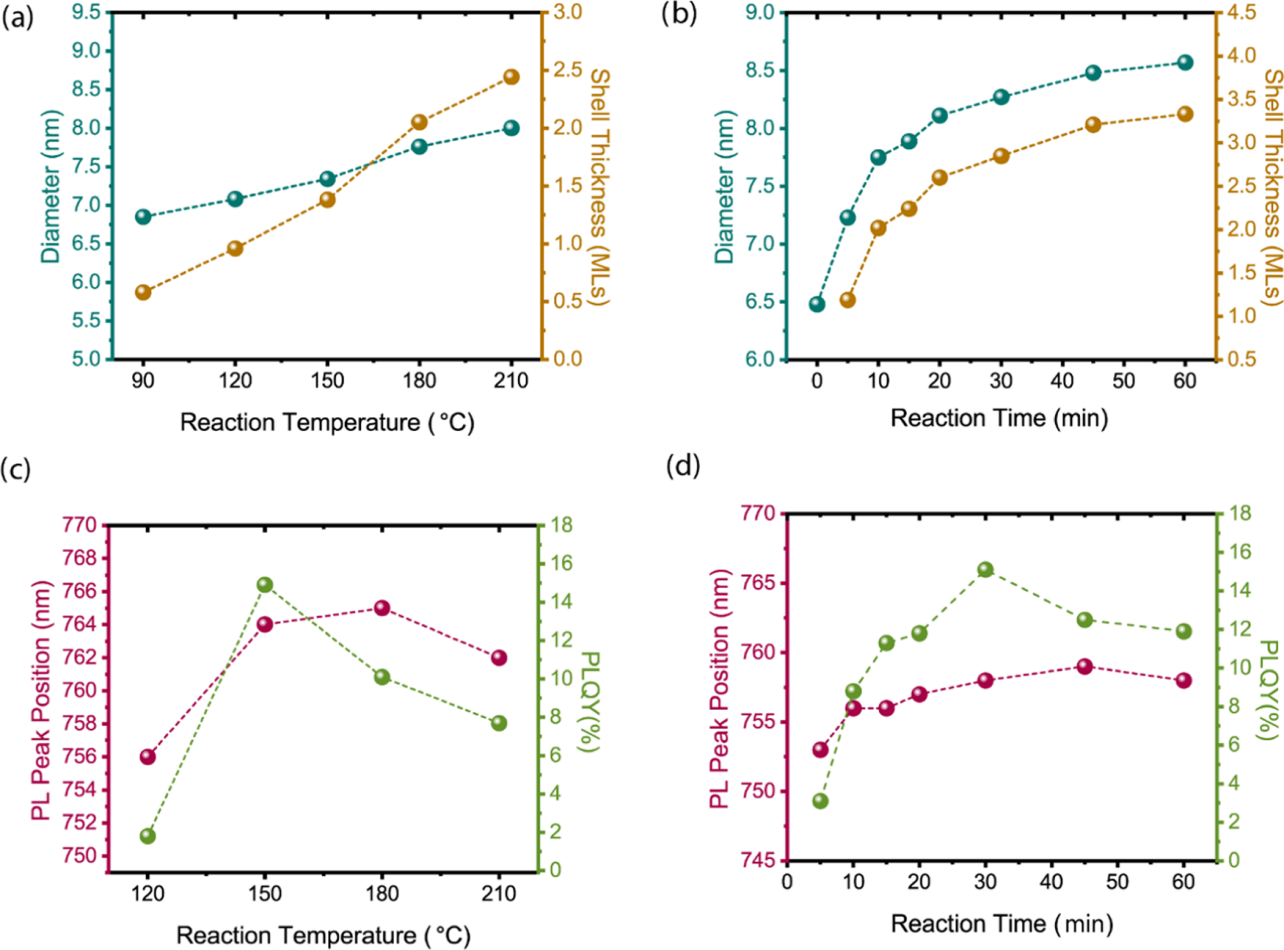
Effect of the (a) reaction temperature and (b) time on the size and shell thickness of the AgBiS_2_/ZnS core/shell NCs. The influence of the (c) reaction temperature and (d) time on the PL peak position and PLQY of the AgBiS_2_/ZnS core/shell NCs.

By keeping the optimal Zn/S ratio at 0.6, we investigated the impact of reaction temperature on the structural and optical properties (Figures 5a and S6). As the temperature increases, the shell thickness increases (Figure 5a). At temperatures below 120 °C resulted in no emission due to insufficient precursor decomposition. At 120 °C, a PL peak at 756 nm and a PLQY of 1.8% was observed (Figure 5c). The maximum PLQY of 14.9% (at 764 nm) was achieved at 150 °C with a shell thickness of ∼1.3 MLs (Figure 5a). Beyond 150 °C, PL peak started to blue-shift to the core while dropping the PLQY to 7.7%.

We investigated the influence of reaction time on the structural and optical properties while keeping the optimal Zn/S ratio at 0.6 and temperature at 150 °C (Figure S8). A reaction time of 30 min provided the most efficient NCs with a PL peak at 764 nm and a PLQY of 15.3% (Figure S7). The TEM analysis showed a core size of 8.27 nm and a shell thickness of ∼2.8 monolayers (MLs) (Figure 5b), indicating effective surface coverage (Figure 2f). These results indicated that a reaction temperature of 150 °C and a reaction time of 30 min are optimal for achieving the highest PLQY and preserving the structural integrity of AgBiS_2_/ZnS core/shell NCs. We monitored their PL emission over a 16-day period and observed emission stability above 90% throughout this period (Figure S9). Shorter reaction times (5–20 min) resulted in thinner shells and lower PLQYs. Longer reaction times (45–60 min) led to thicker shells but reduced PLQY possibly due to strain-induced defect formations (Figure 5d).^65^

## Discussion and Conclusions

AgBiS_2_ NCs offer a promising, eco-friendly alternative to traditional lead- and cadmium-based QDs.^66^ Made from nontoxic and earth-abundant elements, AgBiS_2_ NCs support environmental sustainability and cost efficiency.^67^ Their high absorption coefficient (>10^5^ cm^–1^) across the visible to near-infrared spectrum allows for the creation of thin, flexible, and highly effective photoactive layers.^13^ These advantages make AgBiS_2_ NCs suitable for advanced solar energy harvesting, optical sensing, and photodynamic therapy.^68−70^ The introduction of light-emission via shell growth can enable their use for light-emitting diodes and fluorescent tags.

To synthesize AgBiS_2_ NCs suitable for ZnS shell growth, we used elemental sulfur dissolved in oleylamine (S-OLA) instead of the more reactive bis(trimethylsilyl) sulfide ((TMS)_2_S). S-OLA was chosen as the sulfur source because it enables to synthesize highly crystalline and stable core NCs, which are essential for uniform and stable shell formation. Using S-OLA at 180 °C improved the crystallinity, uniformity, and size distribution of the AgBiS_2_ NCs (Figure 1b–d). Better crystallinity simultaneously reduces surface defects and improves structural stability, both of which are beneficial for ZnS shell growth. Additionally, oleylamine in S-OLA stabilizes the surface of the core NC and improves compatibility with Zn precursors, making shell growth more uniform. In contrast, (TMS)_2_S rapidly releases sulfur at lower temperatures (∼100 °C), and it leads to less stable core NCs and uncontrolled shell formation. Hence, S-OLA enabled high-quality AgBiS_2_ cores that support successful ZnS shell growth.

The asymmetric PL profile, characterized by a pronounced low-energy tail, is primarily attributed to self-absorption effects^71,72^ and the presence of surface traps^73^ at the core/shell interface. Self-absorption reshapes the emission spectrum by selectively reabsorbing high-energy photons and re-emitting at lower energies that enhance the low-energy tail of PL. Surface traps may introduce additional low-energy states leading to radiative recombination below the primary excitonic energy and asymmetrically broadening the PL spectrum. To further validate the structural integrity of the AgBiS_2_/ZnS core/shell NCs and to confirm the origin of their emission, we conducted a series of control syntheses (see Supporting Information (SI)). Based on the results of these control experiments, we conclude that the observed emission originates from the AgBiS_2_ core following the growth of the ZnS shell. No contributions to the emission from byproducts were detected.

The excitonic Bohr radius is a critical parameter in QD systems, representing the average distance between the electron and hole in an exciton. When the core size of a QD is below this radius, quantum confinement effect starts to be observed.^74−76^ AgBiS_2_, however, possesses an indirect bandgap,^77−79^ which typically results in inefficient exciton formation, thus a lack of a pronounced excitonic absorption peak (Figure S7) and have low PLQY. Other indirect bandgap materials, such as silicon,^80^ share a similar characteristic and exhibits very low PLQY (<0.01% in the bulk form).^81^ Nonetheless, silicon nanostructures can achieve high PLQY levels without any shell.^82,83^ Differently, we did not observe any emission from AgBiS_2_ NCs even for smaller sizes. The notable anharmonicity of the Bi–S bond in cubic AgBiS_2_ NCs, along with the disorder in Ag and Bi atom positions,^14,84,85^ may induce enhanced phonon–phonon interactions, further limiting their emission in their nanoscale forms below the Bohr exciton radius. Formation of the ZnS shell around AgBiS_2_ led to a substantial increase in PLQY likely due to strain-induced bright exciton states^86^ and effective passivation of surface trap states, which may also facilitate an indirect-to-direct bandgap transition and enhance radiative recombination.^87^

In conclusion, our study successfully demonstrated the formation of AgBiS_2_/ZnS core/shell NCs, leading to the first-time observation of photoluminescence from the AgBiS_2_ NCs. The improvement of the crystallinity, shape, and uniformity of the AgBiS_2_ core NCs combined with the determination of the proper zinc precursor enabled the ZnS shell growth that led to a photoluminescence at 764 nm with a PLQY of 15.3%. The nontoxic and earth-abundant composition of the NCs points out a new economically feasible and environmentally sustainable light-emitting nanomaterial. Beyond the investigated shell, there exist a wide range of shell options (ZnSe, ZnSeS, InP, GaP) that can further boost their emission efficiency. We consider that light-emitting character of AgBiS_2_ NCs in conjunction with ultrastrong absorption show high promise for future lighting, display, and bioimaging applications.

## Materials and Methods

### Chemicals

Silver acetate (Ag(OAc), 99.99%, powder), bismuth acetate (Bi(OAc)_3_, ≥99.99%, powder), sulfur (S, 99.998%, powder), Bis(trimethylsilyl) sulfide ((TMS)_2_S, synthesis grade), oleic acid (OA, technical grade, 90%), 1-octadecene (ODE, 90%), oleylamine (OLA, ≥98%), zinc acetylacetonate (Zn(acac)_2_, 99.995%, powder), zinc acetate dihydrate (Zn(ac)_2_, 99.999%, powder), 1-dodecanethiol (≥98%) (DDT), were purchased from Sigma-Aldrich. These chemicals were used without further purification, apart from OLA. The OLA was degassed for 8 h at 80 °C and subsequently stored in a nitrogen-filled glovebox.

### Synthesis of (TMS)_2_S Based AgBiS_2_ Core NCs

For the synthesis of AgBiS_2_ core NCs, 1 mmol of Bi(OAc)_3_, 0.8 mmol of Ag(OAc), 5.4 mL of OA, and 2.6 mL of ODE were stirred under vacuum at 100 °C for 2 h to form Bi and Ag oleates and to remove oxygen and moisture. The reaction atmosphere was then switched to argon, and 1 mmol of (TMS)_2_S dissolved in 5 mL of ODE was swiftly injected into the flask. The heating was immediately stopped by removing the heating mantle, and the reaction mixture was rapidly cooled using a cold-water bath. The reaction was then allowed to proceed with stirring at room temperature for 1 h. The NCs were isolated by adding acetone, followed by centrifugation, and purified in toluene. Finally, the solution was filtered using a 0.45 μm pore-sized syringe filter for further characterization.

### Preparation of Ag–Bi Oleate Precursor (for S-OLA Based AgBiS_2_ NCs)

3 mmol of Ag(OAc), 1.5 mmol of Bi(OAc)_3_, 6 mL of OA, and 10 mL of ODE were degassed in a 100 mL three-neck round-bottom flask at 70 °C for 2 h. Following degassing, the temperature was raised to 140 °C under an argon atmosphere and maintained at this temperature for the injection process.

### Synthesis of S-OLA Based AgBiS_2_ Core NCs

AgBiS_2_ core NCs were synthesized as follows. 3 mmol of S and 3 mL of OLA were combined in a 100 mL three-neck round-bottom flask. Under an inert atmosphere, the mixture was heated to 100 °C and maintained under vacuum for 2 h to degas the solution. Following degassing, the temperature was increased to 180 °C under an argon flow. Once the temperature stabilized, 8 mL of an Ag–Bi oleate precursor was injected into the solution. The reaction mixture was maintained at 180 °C for an additional 20 min before the reaction flask was allowed to cool to room temperature. The NCs were separated by adding ethanol to the mixture, followed by centrifugation. They were then purified using *n*-hexane as the solvent. The final solution was filtered through a syringe filter with a 0.45 μm pore size for subsequent characterization.

### Preparation of Zn Precursors for ZnS Shell

0.19 mmol of Zn(acac)_2_ was dissolved in a solution consisting of 1.2 mL of OA and 1.2 mL of ODE at a temperature of 100 °C. Subsequently, 1.6 mL of this prepared solution was extracted for the injection process. Other zinc source was prepared using the same molar amount (0.19 mmol) under identical conditions.

### Preparation of S Precursors for ZnS Shell

Elemental sulfur (0.25 mmol) was dissolved in 1.2 mL of OLA at a temperature of 80 °C. Subsequently, 1 mL of this prepared solution was extracted for the injection process.

For DDT based sulfur sources, 0.25 mmol of DDT was mixed in 1.2 mL of ODE, and 1 mL of this solution was extracted for injection.

### Synthesis of AgBiS_2_/ZnS Core/Shell NCs

The ZnS overcoating on AgBiS_2_ core NCs was carried out using a hot injection approach. In a typical procedure, 2 mL of OLA, 2 mL of OA, and 3 mL of ODE were combined in a 100 mL reaction flask. The solution was degassed at 100 °C for 1 h. Subsequently, the reaction mixture was cooled to 60 °C, and 0.13 mmol of AgBiS_2_ core NCs was injected into the flask. The mixture was then subjected to vacuum for 1 h. Following degassing, the reaction temperature was increased to 150 °C under an argon flow. The prepared zinc and sulfur precursors were then sequentially injected into the reaction mixture. The reaction was allowed to proceed for 30 min before being cooled to room temperature. For purification, the NCs were precipitated and redispersed using ethanol and *n*-hexane, respectively.

### Optical and Structural Characterizations

X-ray powder diffraction (XRD) measurements for AgBiS_2_ core and AgBiS_2_/ZnS core/shell NCs were carried out using a Rigaku MiniFlex 600 diffractometer, equipped with Cu Kα radiation (λ = 1.54 Å). The diffraction patterns were acquired over a 2θ range between 20 and 60° at a scanning rate of 5° per minute. The XRD samples were created by placing NCs dissolved in *n*-hexane onto 1 cm × 1 cm silicon wafers. These were then heated to 70 °C and kept at that temperature for 30 min to ensure the NCs were completely dried. Ultraviolet (UV)–visible absorption spectra of the NCs were tested using an Edinburgh Instruments Spectrofluorometer FS5 with an excitation monochromator. PL and PLQY measurements were conducted with an Edinburgh FLS1000 spectrofluorometer equipped with an integrating sphere. The samples were diluted in *n*-hexane until the optical density at 610 nm wavelength reached 0.1 for UV–visible absorbance. A xenon lamp, set to excitation wavelengths of 310 and 610 nm, was employed for the PL and PLQY measurements, respectively. Time-dependent photoluminescence (PL) decay measurements were performed using a PicoQuant MicroTime 100 time-resolved confocal fluorescence microscope. Excitation was achieved with a 375 nm picosecond diode laser operating at a 10 MHz repetition rate, coupled to a 40× objective lens. Spectrally resolved data were collected using 594 nm long-pass filters. X-ray photoelectron spectroscopy (XPS) measurements were conducted using a Thermo Scientific K-α instrument equipped with an Al Kα source, under ultrahigh vacuum conditions. The XPS samples were prepared following the same protocol used for XRD sample preparation. For TEM measurements, 10 μL of a 1 mM nanocrystal (NC) solution in hexane was deposited onto a copper support grid. TEM, high-resolution TEM (HRTEM), and high-angle annular dark-field scanning TEM (HAADF-STEM) analyses were conducted using a HITACHI HF5000 200 kV (S)TEM.

## Data Availability

The data sets that support all the findings of this study are available from the corresponding authors upon reasonable request.

## Supporting Information Available

Synthesis; HR-TEM; XPS; HAADF-STEM; absorbance; photoluminescence spectra; TEM; PL stability; PLQY; PL decays; XRD (PDF)

## References

[ref1] Pietryga J. M.; Park Y. S.; Lim J.; Fidler A. F.; Bae W. K.; Brovelli S.; Klimov V. I. Spectroscopic and Device Aspects of Nanocrystal Quantum Dots. Chem. Rev. 2016, 116 (18), 10513–10622. 10.1021/acs.chemrev.6b00169. 27677521

[ref2] Carey G. H.; Abdelhady A. L.; Ning Z.; Thon S. M.; Bakr O. M.; Sargent E. H. Colloidal Quantum Dot Solar Cells. Chem. Rev. 2015, 115 (23), 12732–12763. 10.1021/acs.chemrev.5b00063. 26106908

[ref3] Algar W. R.; Massey M.; Rees K.; Higgins R.; Krause K. D.; Darwish G. H.; Peveler W. J.; Xiao Z.; Tsai H. Y.; Gupta R.; Lix K.; Tran M. V.; Kim H. Photoluminescent Nanoparticles for Chemical and Biological Analysis and Imaging. Chem. Rev. 2021, 121 (15), 9243–9358. 10.1021/acs.chemrev.0c01176. 34282906

[ref4] Sadeghi S.; Kumar B. G.; Melikov R.; Aria M. M.; Jalali H. B.; Nizamoglu S. Quantum Dot White LEDs with High Luminous Efficiency. Optica 2018, 5 (7), 793–802. 10.1364/OPTICA.5.000793.

[ref5] Kang C.; Prodanov M. F.; Gao Y.; Mallem K.; Yuan Z.; Vashchenko V. V.; Srivastava A. K. Quantum-Rod On-Chip LEDs for Display Backlights with Efficacy of 149 Lm W–1: A Step toward 200 Lm W–1. Adv. Mater. 2021, 33, 2104685 10.1002/adma.202104685. 34632633

[ref6] Guzelturk B.; Kelestemur Y.; Gungor K.; Yeltik A.; Akgul M. Z.; Wang Y.; Chen R.; Dang C.; Sun H.; Demir H. V. Stable and Low-Threshold Optical Gain in CdSe/CdS Quantum Dots: An All-Colloidal Frequency Up-Converted Laser. Adv. Mater. 2015, 27 (17), 2741–2746. 10.1002/adma.201500418. 25807924

[ref7] Yu W. W.; Qu L.; Guo W.; Peng X. Experimental Determination of the Extinction Coefficient of CdTe, CdSe and CdS Nanocrystals. Chem. Mater. 2004, 16, 2854–2860. 10.1021/cm033007z.

[ref8] Lin Q.; Yun H. J.; Liu W.; Song H. J.; Makarov N. S.; Isaienko O.; Nakotte T.; Chen G.; Luo H.; Klimov V. I.; Pietryga J. M. Phase-Transfer Ligand Exchange of Lead Chalcogenide Quantum Dots for Direct Deposition of Thick, Highly Conductive Films. J. Am. Chem. Soc. 2017, 139 (19), 6644–6653. 10.1021/jacs.7b01327. 28431206

[ref9] Gu K.; Wu H.; Su J.; Sun P.; Tan P.-H.; Zhong H. Size Dependent Specific Heat Capacity of PbSe Nanocrystals. Nano Lett. 2024, 24 (13), 4038–4043. 10.1021/acs.nanolett.4c01021. 38511834

[ref10] Lifshitz E.; Bashouti M.; Kloper V.; Kigel A.; Eisen M. S.; Berger S. Synthesis and Characterization of PbSe Quantum Wires, Multipods, Quantum Rods, and Cubes. Nano Lett. 2003, 3 (6), 857–862. 10.1021/nl0342085.

[ref11] Bernechea M.; Miller N. C.; Xercavins G.; So D.; Stavrinadis A.; Konstantatos G. Solution-Processed Solar Cells Based on Environmentally Friendly AgBiS2 Nanocrystals. Nat. Photonics 2016, 10 (8), 521–525. 10.1038/nphoton.2016.108.

[ref12] Ajiboye T. O.; Mafolasire A. A.; Lawrence S.; Tyhali N.; Mhlanga S. D. Composite and Pristine Silver Bismuth Sulphide: Synthesis and Up-to-Date Applications. J. Inorg. Organomet. Polym. Mater. 2024, 34 (2), 433–457. 10.1007/s10904-023-02838-y.

[ref13] Wang Y.; Kavanagh S. R.; Burgués-Ceballos I.; Walsh A.; Scanlon D.; Konstantatos G. Cation Disorder Engineering Yields AgBiS2 Nanocrystals with Enhanced Optical Absorption for Efficient Ultrathin Solar Cells. Nat. Photonics 2022, 16 (3), 235–241. 10.1038/s41566-021-00950-4.

[ref14] Guin S. N.; Biswas K. Cation Disorder and Bond Anharmonicity Optimize the Thermoelectric Properties in Kinetically Stabilized Rocksalt AgBiS2 Nanocrystals. Chem. Mater. 2013, 25 (15), 3225–3231. 10.1021/cm401630d.

[ref15] Treharne R. E.; Seymour-Pierce A.; Durose K.; Hutchings K.; Roncallo S.; Lane D. Optical Design and Fabrication of Fully Sputtered CdTe/CdS Solar Cells. J. Phys.:Conf. Ser. 2011, 286, 012038 10.1088/1742-6596/286/1/012038.

[ref16] Massiot I.; Cattoni A.; Collin S. Progress and Prospects for Ultrathin Solar Cells. Nat. Energy 2020, 5 (12), 959–972. 10.1038/s41560-020-00714-4.

[ref17] Palik E. D. Handbook of Optical Constants of Solids; Academic Press, 1998; Vol. 3.

[ref18] Jalali H. B.; Sadeghi S.; Yuksel I. B. D.; Onal A.; Nizamoglu S. Past, Present and Future of Indium Phosphide Quantum Dots. Nano Res. 2022, 15, 4468–4489. 10.1007/s12274-021-4038-z.

[ref19] Manzoor S.; Häusele J.; Bush K. A.; Palmstrom A. F.; Carpenter J. III; Yu Z. J.; Bent S. F.; Mcgehee M. D.; Holman Z. C. Optical Modeling of Wide-Bandgap Perovskite and Perovskite/Silicon Tandem Solar Cells Using Complex Refractive Indices for Arbitrary-Bandgap Perovskite Absorbers. Opt. Express 2018, 26 (21), 27441–27460. 10.1364/OE.26.027441. 30469811

[ref20] Cheng J.; Wang W.; Xu X.; Lin Z.; Xie C.; Zhang Y.; Zhang T.; Li L.; Lu Y.; Li Q. AgBiS2 Nanoparticles with Synergistic Photodynamic and Bioimaging Properties for Enhanced Malignant Tumor Phototherapy. Mater. Sci. Eng.: C 2020, 107, 110324 10.1016/j.msec.2019.110324. 31761161

[ref21] Burgués-Ceballos I.; Wang Y.; Akgul M. Z.; Konstantatos G. Colloidal AgBiS2 Nanocrystals with Reduced Recombination Yield 6.4% Power Conversion Efficiency in Solution-Processed Solar Cells. Nano Energy 2020, 75, 104961 10.1016/j.nanoen.2020.104961.

[ref22] Balamur R.; Oh J. T.; Karatum O.; Wang Y.; Onal A.; Kaleli H. N.; Pehlivan C.; Şahin A.; Hasanreisoglu M.; Konstantatos G.; Nizamoglu S. Capacitive and Efficient Near-Infrared Stimulation of Neurons via an Ultrathin AgBiS2 Nanocrystal Layer. ACS Appl. Mater. Interfaces 2024, 16 (23), 29610–29620. 10.1021/acsami.4c01964. 38807565 PMC11661670

[ref23] Selopal G. S.; Zhao H.; Wang Z. M.; Rosei F. Core/Shell Quantum Dots Solar Cells. Adv. Funct. Mater. 2020, 30 (13), 1908762 10.1002/adfm.201908762.

[ref24] Yang J.; Wang J.; Zhao K.; Izuishi T.; Li Y.; Shen Q.; Zhong X. CdSeTe/CdS Type-I Core/Shell Quantum Dot Sensitized Solar Cells with Efficiency over 9%. J. Phys. Chem. C 2015, 119 (52), 28800–28808. 10.1021/acs.jpcc.5b10546.

[ref25] Ning Z.; Tian H.; Yuan C.; Fu Y.; Qin H.; Sun L.; Ågren H. Solar Cells Sensitized with Type-II ZnSe–CdS Core/Shell Colloidal Quantum Dots. Chem. Commun. 2011, 47 (5), 1536–1538. 10.1039/C0CC03401K. 21103496

[ref26] Chaudhuri R. G.; Paria S. Core/Shell Nanoparticles: Classes, Properties, Synthesis Mechanisms, Characterization, and Applications. Chem. Rev. 2012, 112 (4), 2373–2433. 10.1021/cr100449n. 22204603

[ref27] Bonifacio C. S.; Carenco S.; Wu C. H.; House S. D.; Bluhm H.; Yang J. C. Thermal Stability of Core–Shell Nanoparticles: A Combined in Situ Study by XPS and TEM. Chem. Mater. 2015, 27 (20), 6960–6968. 10.1021/acs.chemmater.5b01862.

[ref28] Tamang S.; Lincheneau C.; Hermans Y.; Jeong S.; Reiss P. Chemistry of InP Nanocrystal Syntheses. Chem. Mater. 2016, 28 (8), 2491–2506. 10.1021/acs.chemmater.5b05044.

[ref29] Akgul M. Z.; Konstantatos G. AgBiSe2Colloidal Nanocrystals for Use in Solar Cells. ACS Appl. Nano Mater. 2021, 4 (3), 2887–2894. 10.1021/acsanm.1c00048.

[ref30] Dong J.; Zhang D.; Liu J.; Jiang Y.; Tan X. Y.; Jia N.; Cao J.; Suwardi A.; Zhu Q.; Xu J.; Li J. F.; Yan Q. N-Type Thermoelectric AgBiPbS3 with Nanoprecipitates and Low Thermal Conductivity. Inorg. Chem. 2023, 62 (43), 17905–17912. 10.1021/acs.inorgchem.3c02777. 37843461

[ref31] Guin S. N.; Banerjee S.; Sanyal D.; Pati S. K.; Biswas K. Nanoscale Stabilization of Nonequilibrium Rock Salt BiAgSeS: Colloidal Synthesis and Temperature Driven Unusual Phase Transition. Chem. Mater. 2017, 29 (8), 3769–3777. 10.1021/acs.chemmater.7b00862.

[ref32] Paul S.; Dalal B.; Jana R.; Shit A.; Datta A.; De S. K. Enhanced Photophysical Properties of Bi2S3/AgBiS2Nanoheterostructures Synthesized via Ag(I) Cation Exchange-Mediated Transformation of Binary Bi2S3. J. Phys. Chem. C 2020, 124 (23), 12824–12833. 10.1021/acs.jpcc.0c03487.

[ref33] Geller S.; Wernick J. H. Ternary Semiconducting Compounds with Sodium Chloride-like Structure: AgSbSe2, AgSbTe2, AgBiS2, AgBiSe2. Acta Crystallogr. 1959, 12 (1), 46–54. 10.1107/S0365110X59000135.

[ref34] Jumpertz E. A. Ueber Die Elektronendichteverteilung in Der Zinkblende. Z. Elektrochem., Ber. Bunsenges. Phys. Chem. 1955, 59 (5), 419–425. 10.1002/bbpc.19550590520.

[ref35] Hao J. J.; Zhou J.; Zhang C. Y. A Tri-n-Octylphosphine-Assisted Successive Ionic Layer Adsorption and Reaction Method to Synthesize Multilayered Core–Shell CdSe–ZnS Quantum Dots with Extremely High Quantum Yield. Chem. Commun. 2013, 49 (56), 6346–6348. 10.1039/c3cc43147a. 23748410

[ref36] Jiang P.; Zhu C. N.; Zhu D. L.; Zhang Z. L.; Zhang G. J.; Pang D. W. A Room-Temperature Method for Coating a ZnS Shell on Semiconductor Quantum Dots. J. Mater. Chem. C 2015, 3 (5), 964–967. 10.1039/C4TC02437K.

[ref37] Jiang P.; Wang R.; Chen Z. Thiol-Based Non-Injection Synthesis of near-Infrared Ag2S/ZnS Core/Shell Quantum Dots. RSC Adv. 2015, 5 (70), 56789–56793. 10.1039/C5RA08008H.

[ref38] Saha A.; Figueroba A.; Konstantatos G. Ag2ZnSnS4 Nanocrystals Expand the Availability of RoHS Compliant Colloidal Quantum Dots. Chem. Mater. 2020, 32 (5), 2148–2155. 10.1021/acs.chemmater.9b05370.

[ref39] Saha A.; Konstantatos G. Ag2ZnSnS4-ZnS Core-Shell Colloidal Quantum Dots: A near-Infrared Luminescent Material Based on Environmentally Friendly Elements. J. Mater. Chem. C 2021, 9 (17), 5682–5688. 10.1039/D1TC00421B. PMC8101413 33996096

[ref40] Deng D.; Chen Y.; Cao J.; Tian J.; Qian Z.; Achilefu S.; Gu Y. High-Quality CuInS 2/ZnS Quantum Dots for in Vitro and in Vivo Bioimaging. Chem. Mater. 2012, 24 (15), 3029–3037. 10.1021/cm3015594.

[ref41] Berends A. C.; Van Der Stam W.; Hofmann J. P.; Bladt E.; Meeldijk J. D.; Bals S.; De Mello Donega C. Interplay between Surface Chemistry, Precursor Reactivity, and Temperature Determines Outcome of ZnS Shelling Reactions on CuInS2 Nanocrystals. Chem. Mater. 2018, 30 (7), 2400–2413. 10.1021/acs.chemmater.8b00477. 29657360 PMC5895981

[ref42] Shen H.; Wang H.; Tang Z.; Niu J. Z.; Lou S.; Du Z.; Li L. S. High Quality Synthesis of Monodisperse Zinc-Blende CdSe and CdSe/ZnS Nanocrystals with a Phosphine-Free Method. CrystEngComm 2009, 11 (8), 1733–1738. 10.1039/b909063k.

[ref43] Li Q.; Zheng X.; Shen X.; Ding S.; Feng H.; Wu G.; Zhang Y. Optimizing the Synthetic Conditions of “Green” Colloidal AgBiS2 Nanocrystals Using a Low-Cost Sulfur Source. Nanomaterials 2022, 12 (21), 3742 10.3390/nano12213742. 36364517 PMC9654632

[ref44] Wang Y.; Peng L.; Wang Z.; Konstantatos G. Environmentally Friendly AgBiS2 Nanocrystal Inks for Efficient Solar Cells Employing Green Solvent Processing. Adv. Energy Mater. 2022, 12 (21), 2200700 10.1002/aenm.202200700.

[ref45] Yuan M.; Kemp K. W.; Thon S. M.; Kim J. Y.; Chou K. W.; Amassian A.; Sargent E. H. High-Performance Quantum-Dot Solids via Elemental Sulfur Synthesis. Adv. Mater. 2014, 26 (21), 3513–3519. 10.1002/adma.201305912. 24659303

[ref46] Park J.; Jayaraman A.; Schrader A. W.; Hwang G. W.; Han H.-S. Controllable Modulation of Precursor Reactivity Using Chemical Additives for Systematic Synthesis of High-Quality Quantum Dots. Nat. Commun. 2020, 11 (1), 5748 10.1038/s41467-020-19573-4. 33184282 PMC7665041

[ref47] Mourdikoudis S.; Menelaou M.; Fiuza-Maneiro N.; Zheng G.; Wei S.; Pérez-Juste J.; Polavarapu L.; Sofer Z. Oleic Acid/Oleylamine Ligand Pair: A Versatile Combination in the Synthesis of Colloidal Nanoparticles. Nanoscale Horiz. 2022, 7 (9), 941–1015. 10.1039/D2NH00111J. 35770698

[ref48] Dabbousi B. O.; Rodriguez-Viejo J.; Mikulec F. V.; Heine J. R.; Mattoussi H.; Ober R.; Jensen K. F.; Bawendi M. G. CdSe) ZnS Core– Shell Quantum Dots: Synthesis and Characterization of a Size Series of Highly Luminescent Nanocrystallites. J. Phys. Chem. B 1997, 101 (46), 9463–9475. 10.1021/jp971091y.

[ref49] Kang X.; Huang L.; Yang Y.; Pan D. Scaling up the Aqueous Synthesis of Visible Light Emitting Multinary AgInS2/ZnS Core/Shell Quantum Dots. J. Phys. Chem. C 2015, 119 (14), 7933–7940. 10.1021/acs.jpcc.5b00413.

[ref50] Manimozhi T.; Kavirajan S.; Bharathi K. K.; Kumar E. S.; Navaneethan M. Ultra-Low Thermal Conductivity of AgBiS2 via Sb Substitution as a Scattering Center for Thermoelectric Applications. J. Mater. Sci.: Mater. Electron. 2022, 33 (16), 12615–12628. 10.1007/s10854-022-08211-y.

[ref51] Tabatabaei K.; Lu H.; Nolan B. M.; Cen X.; McCold C. E.; Zhang X.; Brutchey R. L.; van Benthem K.; Hihath J.; Kauzlarich S. M. Bismuth Doping of Germanium Nanocrystals through Colloidal Chemistry. Chem. Mater. 2017, 29 (17), 7353–7363. 10.1021/acs.chemmater.7b02241.

[ref52] Khot K. V.; Mali S. S.; Pawar N. B.; Kharade R. R.; Mane R. M.; Kondalkar V. V.; Patil P. B.; Patil P. S.; Hong C. K.; Kim J. H.; Heo J.; Bhosale P. N. Development of Nanocoral-like Cd(SSe) Thin Films Using an Arrested Precipitation Technique and Their Application. New J. Chem. 2014, 38 (12), 5964–5974. 10.1039/C4NJ01319K.

[ref53] Chen W.; Wang W.; Sun L.; Chen S.; Yan Q.; Guo T.; Zhou X.; Wu C.; Zhang Y. Synthesis and Characterization of InP/ZnSe/ZnS Quantum Dots for Photo-Emissive Color Conversion. Opt. Mater. Express 2022, 12 (4), 1717–1730. 10.1364/OME.453712.

[ref54] Ka̧kołowicz W.; Giera E. Standard Enthalpies of Formation of the Chelate Complexes of Some 3d-Electron Elements with Pentane-2,4-Dione Metal-Oxygen Bond Energies and Ligand-Field Stabilization Energies. J. Chem. Thermodyn. 1983, 15 (3), 203–210. 10.1016/0021-9614(83)90109-X.

[ref55] Hughes J. T.; Navrotsky A. Enthalpy of Formation of Zinc Acetate Dihydrate. J. Chem. Thermodyn. 2011, 43 (6), 980–982. 10.1016/j.jct.2011.02.004.

[ref56] Lide D. R. CRC Handbook of Chemistry and Physics; CRC Press, 2004; Vol. 85.

[ref57] Rappoport Z.; Marek I. The Chemistry of Organomagnesium Compounds; John Wiley & Sons, 2008.

[ref58] Thomson J. W.; Nagashima K.; MacDonald P. M.; Ozin G. A. From Sulfur-Amine Solutions to Metal Sulfide Nanocrystals: Peering into the Oleylamine-Sulfur Black Box. J. Am. Chem. Soc. 2011, 133 (13), 5036–5041. 10.1021/ja1109997. 21384888

[ref59] Hao J.; Liu H.; Miao J.; Lu R.; Zhou Z.; Zhao B.; Xie B.; Cheng J.; Wang K.; Delville M.-H. A Facile Route to Synthesize CdSe/ZnS Thick-Shell Quantum Dots with Precisely Controlled Green Emission Properties: Towards QDs Based LED Applications. Sci. Rep. 2019, 9 (1), 12048 10.1038/s41598-019-48469-7. 31427624 PMC6700096

[ref60] Won Y. H.; Cho O.; Kim T.; Chung D. Y.; Kim T.; Chung H.; Jang H.; Lee J.; Kim D.; Jang E. Highly Efficient and Stable InP/ZnSe/ZnS Quantum Dot Light-Emitting Diodes. Nature 2019, 575 (7784), 634–638. 10.1038/s41586-019-1771-5. 31776489

[ref61] De Trizio L.; Manna L. Forging Colloidal Nanostructures via Cation Exchange Reactions. Chem. Rev. 2016, 116 (18), 10852–10887. 10.1021/acs.chemrev.5b00739. 26891471 PMC5043423

[ref62] Pons T.; Pic E.; Lequeux N.; Cassette E.; Bezdetnaya L.; Guillemin F.; Marchal F.; Dubertret B. Cadmium-Free CuInS2/ZnS Quantum Dots for Sentinel Lymph Node Imaging with Reduced Toxicity. ACS Nano 2010, 4 (5), 2531–2538. 10.1021/nn901421v. 20387796

[ref63] Yang H.; Zhang L.; Tang Y.; Xiang W.; Wang X.; Xiao M.; Cui Y.; Zhang J. Enhanced Multiexciton Emission Property in Gradient Alloy Core/Shell CdZnSeS/ZnS Quantum Dots: Balance between Surface Passivation and Strain-Induced Lattice Defect. J. Phys. Chem. C 2021, 125 (19), 10759–10767. 10.1021/acs.jpcc.1c02029.

[ref64] Onal A.; Eren G. O.; Melikov R.; Kaya L.; Nizamoglu S. Quantum Dot Enabled Efficient White LEDs for Wide Color Gamut Displays. Adv. Mater. Technol. 2022, 8 (9), 2201799 10.1002/admt.202201799.

[ref65] Ji B.; Koley S.; Slobodkin I.; Remennik S.; Banin U. ZnSe/ZnS Core/Shell Quantum Dots with Superior Optical Properties through Thermodynamic Shell Growth. Nano Lett. 2020, 20 (4), 2387–2395. 10.1021/acs.nanolett.9b05020. 32134676 PMC7467768

[ref66] Jun H. K. Solution-Processed Quantum Dot-Sensitized Solar Cell Based on “Green” Materials. In Sustainable Materials for Next Generation Energy Devices; Elsevier, 2021; pp 133–147.

[ref67] Ju M.-G.; Dai J.; Ma L.; Zhou Y.; Zeng X. C. AgBiS 2 as a Low-Cost and Eco-Friendly All-Inorganic Photovoltaic Material: Nanoscale Morphology–Property Relationship. Nanoscale Adv. 2020, 2 (2), 770–776. 10.1039/C9NA00505F. 36133252 PMC9417815

[ref68] Huang P.-C.; Yang W.-C.; Lee M.-W. AgBiS2 Semiconductor-Sensitized Solar Cells. J. Phys. Chem. C 2013, 117 (36), 18308–18314. 10.1021/jp4046337.

[ref69] Alagumalai K.; Sivakumar M.; Kim S.-C.; Babulal S. M.; Ouladsmane M. AgBiS2 Embedded Activated Graphene Nanolayer for Sensing Azathioprine in Biospecimens. Colloids Surf., A 2024, 685, 133243 10.1016/j.colsurfa.2024.133243.

[ref70] Sun M.; Yang D.; Wang C.; Bi H.; Zhou Y.; Wang X.; Xu J.; He F.; Gai S.; Yang P. AgBiS 2-TPP Nanocomposite for Mitochondrial Targeting Photodynamic Therapy, Photothermal Therapy and Bio-Imaging under 808 Nm NIR Laser Irradiation. Biomater. Sci. 2019, 7 (11), 4769–4781. 10.1039/C9BM01077G. 31509113

[ref71] Liu Y.; Kim D.; Morris O. P.; Zhitomirsky D.; Grossman J. C. Origins of the Stokes Shift in PbS Quantum Dots: Impact of Polydispersity, Ligands, and Defects. ACS Nano 2018, 12 (3), 2838–2845. 10.1021/acsnano.8b00132. 29513986

[ref72] Cai W.; Ren Y.; Huang Z.; Sun Q.; Shen H.; Wang Y. Emission Mechanism of Bright and Eco-Friendly ZnSeTe Quantum Dots. Adv. Opt. Mater. 2024, 12 (6), 2301970 10.1002/adom.202301970.

[ref73] Kline J.; Gallagher S.; Hammel B. F.; Mathew R.; Ladd D. M.; Westbrook R. J. E.; Pryor J. N.; Toney M. F.; Pelton M.; Yazdi S. Emissive Surface Traps Lead to Asymmetric Photoluminescence Line Shape in Spheroidal CsPbBr3 Quantum Dots, arXiv:2410.05194. arXiv.org e-Print archive, 2024. https://arxiv.org/abs/2410.05194. 10.1021/acs.nanolett.4c04995 40130575

[ref74] Alivisatos A. P. Semiconductor Clusters, Nanocrystals, and Quantum Dots. Science 1996, 271, 933–937. 10.1126/science.271.5251.933.

[ref75] Bawendi M. G.; Steigerwald M. L.; Brus L. E. The Quantum Mechanics of Larger Semiconductor Clusters (“Quantum Dots”). Annu. Rev. Phys. Chem. 1990, 41 (1), 477–496. 10.1146/annurev.pc.41.100190.002401.

[ref76] Silbey R. J.; Alberty R. A.; Papadantonakis G. A.; Bawendi M. G. Physical Chemistry; John Wiley & Sons, 2022.

[ref77] Ju M. G.; Dai J.; Ma L.; Zhou Y.; Zeng X. C. AgBiS2 as a Low-Cost and Eco-Friendly All-Inorganic Photovoltaic Material: Nanoscale Morphology-Property Relationship. Nanoscale Adv. 2020, 2 (2), 770–776. 10.1039/C9NA00505F. 36133252 PMC9417815

[ref78] Öberg V. A.; Johansson M. B.; Zhang X.; Johansson E. M. J. Cubic AgBiS2Colloidal Nanocrystals for Solar Cells. ACS Appl. Nano Mater. 2020, 3 (5), 4014–4024. 10.1021/acsanm.9b02443.

[ref79] van Embden J.; Gaspera E. D. Ultrathin Solar Absorber Layers of Silver Bismuth Sulfide from Molecular Precursors. ACS Appl. Mater. Interfaces 2019, 11 (18), 16674–16682. 10.1021/acsami.8b22414. 31025846

[ref80] Meinardi F.; Ehrenberg S.; Dhamo L.; Carulli F.; Mauri M.; Bruni F.; Simonutti R.; Kortshagen U.; Brovelli S. Highly Efficient Luminescent Solar Concentrators Based on Earth-Abundant Indirect-Bandgap Silicon Quantum Dots. Nat. Photonics 2017, 11 (3), 177–185. 10.1038/nphoton.2017.5.

[ref81] Xu Y.; Terada S.; Xin Y.; Ueda H.; Saitow K. Ligand Effects on Photoluminescence and Electroluminescence of Silicon Quantum Dots for Light-Emitting Diodes. ACS Appl. Nano Mater. 2022, 5 (6), 7787–7797. 10.1021/acsanm.2c00811.

[ref82] Holmes J. D.; Ziegler K. J.; Doty R. C.; Pell L. E.; Johnston K. P.; Korgel B. A. Highly Luminescent Silicon Nanocrystals with Discrete Optical Transitions. J. Am. Chem. Soc. 2001, 123 (16), 3743–3748. 10.1021/ja002956f. 11457106

[ref83] Hessel C. M.; Reid D.; Panthani M. G.; Rasch M. R.; Goodfellow B. W.; Wei J.; Fujii H.; Akhavan V.; Korgel B. A. Synthesis of Ligand-Stabilized Silicon Nanocrystals with Size-Dependent Photoluminescence Spanning Visible to Near-Infrared Wavelengths. Chem. Mater. 2012, 24 (2), 393–401. 10.1021/cm2032866.

[ref84] Wei B.; Sun Q.; Li C.; Hong J. Phonon Anharmonicity: A Pertinent Review of Recent Progress and Perspective. Sci. China: Phys., Mech. Astron. 2021, 64 (11), 117001 10.1007/s11433-021-1748-7.

[ref85] Rathore E.; Juneja R.; Culver S. P.; Minafra N.; Singh A. K.; Zeier W. G.; Biswas K. Origin of Ultralow Thermal Conductivity in N-Type Cubic Bulk AgBiS 2: Soft Ag Vibrations and Local Structural Distortion Induced by the Bi 6s 2 Lone Pair. Chem. Mater. 2019, 31 (6), 2106–2113. 10.1021/acs.chemmater.9b00001.

[ref86] Zhai J.; Dong T.; Zhou Y.; Min J.; Yan Y.; Garoufalis C. S.; Baskoutas S.; Xu D.; Zeng Z. Efficient Band-Edge Emission from Indirect Bandgap Semiconductor Quantum Dots upon Shell Engineering. Nano Lett. 2023, 23 (8), 3239–3244. 10.1021/acs.nanolett.3c00008. 37022343

[ref87] Shin H.; Hong D.; Cho H.; Jang H.; Kim G. Y.; Song K. M.; Choi M. J.; Kim D.; Jung Y. S. Indirect-to-Direct Bandgap Transition in GaP Semiconductors through Quantum Shell Formation on ZnS Nanocrystals. Nat. Commun. 2024, 15, 8125 10.1038/s41467-024-52535-8. 39284803 PMC11405752

