# Supplementary material for: Emergence of Near-Infrared Photoluminescence via ZnS Shell Growth on the AgBiS2 Nanocrystals
Source: Chem Mater. 2024 Dec 11;37(1):255–65. doi: 10.1021/acs.chemmater.4c02406 (PMC11736682; doi:10.1021/acs.chemmater.4c02406)
Supplement: Supplementary file 1 — cm4c02406_si_001.pdf [file cm4c02406_si_001.pdf]

Supporting Information

Emergence of Near-Infrared Photoluminescence via ZnS Shell Growth on the $AgBiS_2$ Nanocrystals

*Asim Onal[1], Tarık Safa Kaya[2], Önder Metin[3], Sedat Nizamoglu[1, 4, *]*

[1]Graduate School of Biomedical Science and Engineering, Koç University, Istanbul, 34450, Türkiye

[2]Graduate School of Material Science and Engineering, Koç University, Istanbul, 34450, Türkiye

[3]Department of Chemistry, College of Sciences, Koç University, Istanbul, 34450, Türkiye

[4]Department of Electrical and Electronics Engineering, Koç University, Istanbul, 34450, Türkiye

[*]Corresponding Author: snizamoglu@ku.edu.tr

Contents

1. Supporting Data……………………………………………………………………………………………...3

2. Photoluminescence quantum yield & Lifetime ……………………………………………………..13

2.1. Photoluminescence quantum yield (PLQY) of $AgBiS_2$/ZnS NCs……………………………..**13**

2.2. PL decays and PLQY correlation……………………………………………………………………..**15**

3. Control Experiments……………………………………………………………………………………..17

3.1. XRD results of $AgBiS_2$/ZnS NCs at different reaction temperatures………………………..17

3.2. Controlled synthesis of $AgBiS_2$/ZnS nanocrystals: Examining the impact of excluding Zn precursors……………………………………………………………………………………………………18

3.3. Impact of various Ag ratios on $AgBiS_2$ core NCs synthesis: Evaluation of byproduct formation in core/shell structures……………………………………………………………………19

3.4. Control experiment of core/shell NCs synthesis by excluding "Bi" precursor inclusion………20

3.5. Optical absorption and PL spectra of core/shell NCs synthesized by excluding "Bi" precursor inclusion………………………………………………………………………………………………21

3.6. XRD analysis of $AgBiS_2$ core and $AgBiS_2$/ZnS core/shell NCs before and after annealing………22

1. Supporting Data

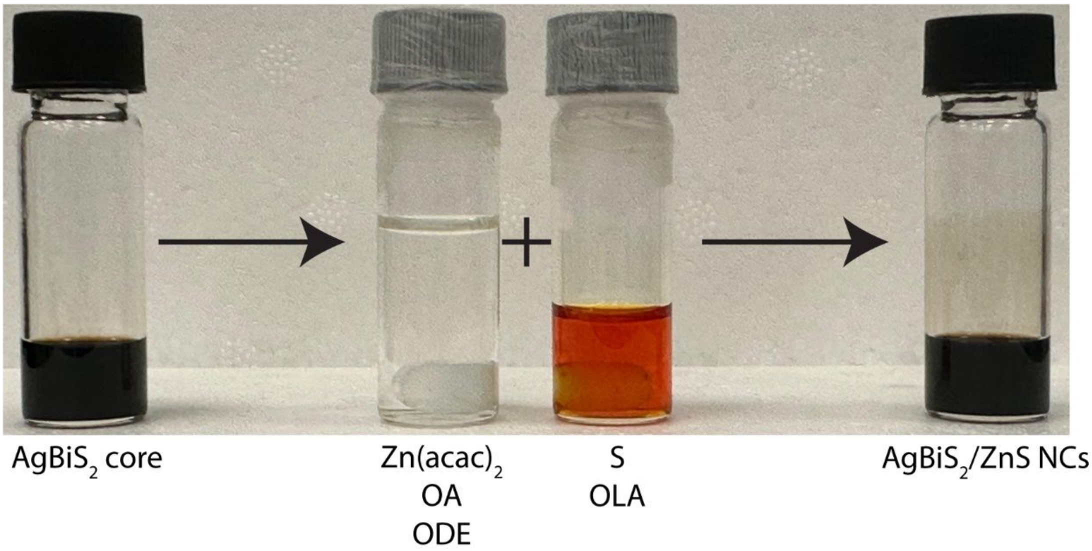

Figure S1. *Photograph of the $AgBiS_2$ core NCs, zinc and sulfur precursors, and the final product of $AgBiS_2$/ZnS core/shell NCs under ambient light.*

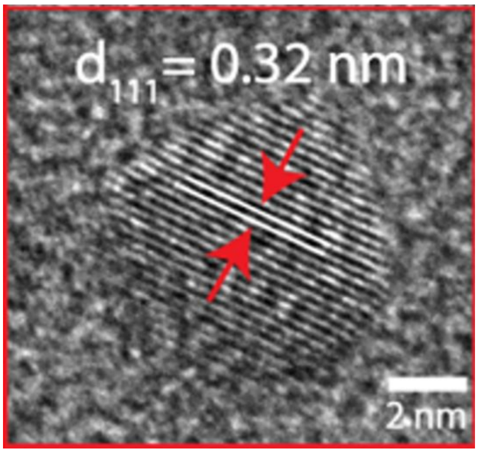

Figure S2. *HRTEM image of the $AgBiS_2$ core NCs synthesized with S-OLA.*

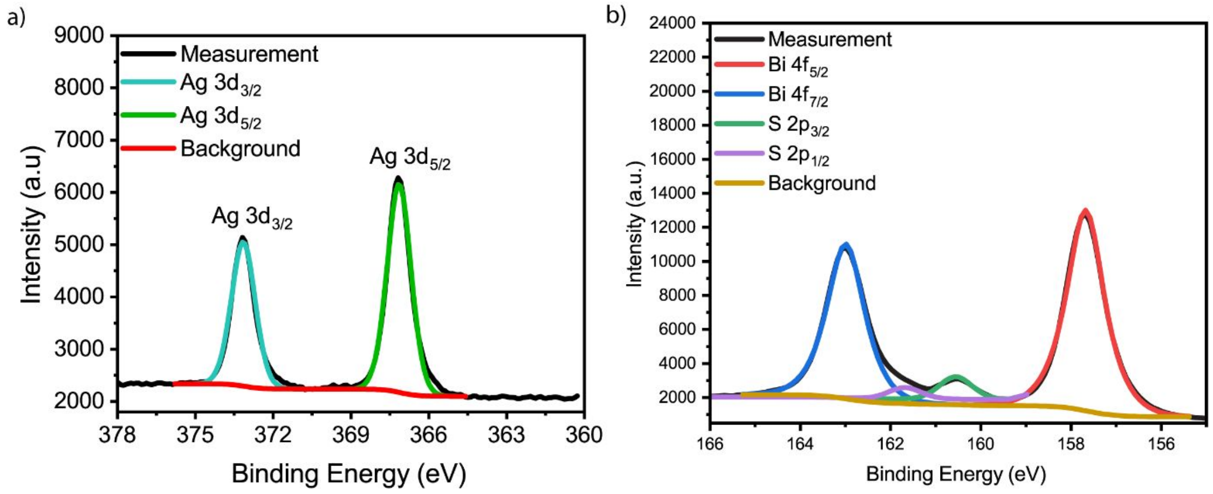

Figure S3. *X-ray photoelectron spectroscopy (XPS) spectra of the synthesized* $AgBiS_2$ *core NCs, displaying the spectra of (a) Ag 3d, (b) Bi 4f and S 2p regions.*

Table S1. *XPS atomic composition (at %) of $AgBiS_2$ core and $AgBiS_2$/ZnS core/shell NCs.*

Sample	XPS Atomic Composition (at %)				
	Ag 3d	Bi 4f	S 2p	Zn 2p	Ag/Bi
$(TMS)_2S$-based $AgBiS_2$ NCs	**30.16**	**44.21**	**25.63**	**-**	**0.68**
S-OLA-based $AgBiS_2$ core NCs	**33.36**	**27.26**	**39.37**	**-**	**1.22**
$AgBiS_2$/ZnS core/shell NCs (Zn/S - 0.3)	**15.22**	**9.43**	**19.5**	**55.85**	**1.61**
$AgBiS_2$/ZnS core/shell NCs (Zn/S - 0.6)	**10.14**	**6.73**	**22.78**	**60.35**	**1.50**
$AgBiS_2$/ZnS core/shell NCs (Zn/S - 0.9)	**8.80**	**5.48**	**24.34**	**61.39**	**1.60**
$AgBiS_2$/ZnS core/shell NCs (Zn/S - 1.2)	**6.67**	**4.86**	**23.85**	**64.61**	**1.37**

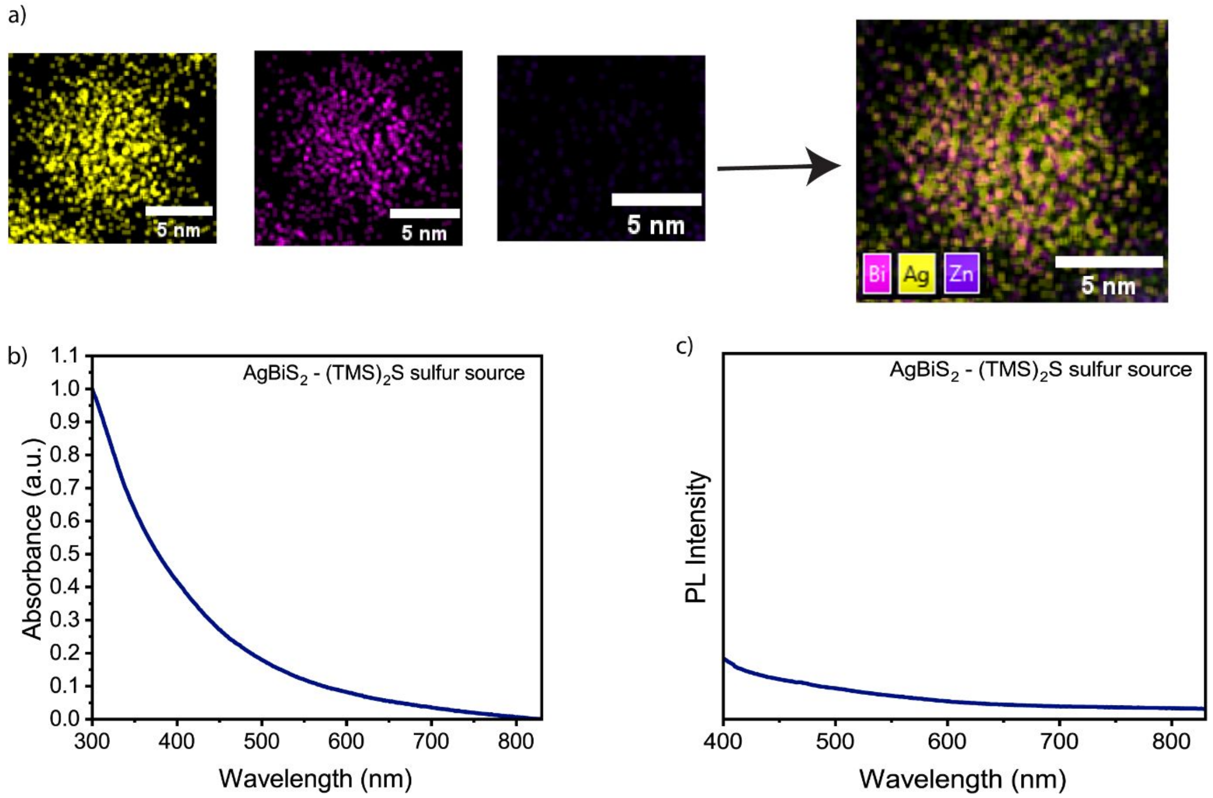

Figure S4. *(a) HAADF-STEM image of $AgBiS_2$/ZnS core/shell NCs via the control synthesis experiment and the corresponding EDX data showing the distributions of Ag, Bi, and Zn along with a superimposed Ag-Bi-Zn element map. It clearly indicates that there is no Zn on the structure. (b) Absorbance spectra of $AgBiS_2$/ZnS core/shell NCs synthesized using $(TMS)_2S$ as the sulfur source for $AgBiS_2$, indicating the presence of $AgBiS_2$. (c) There is no emission by $AgBiS_2$ NCs after ZnS shelling.*

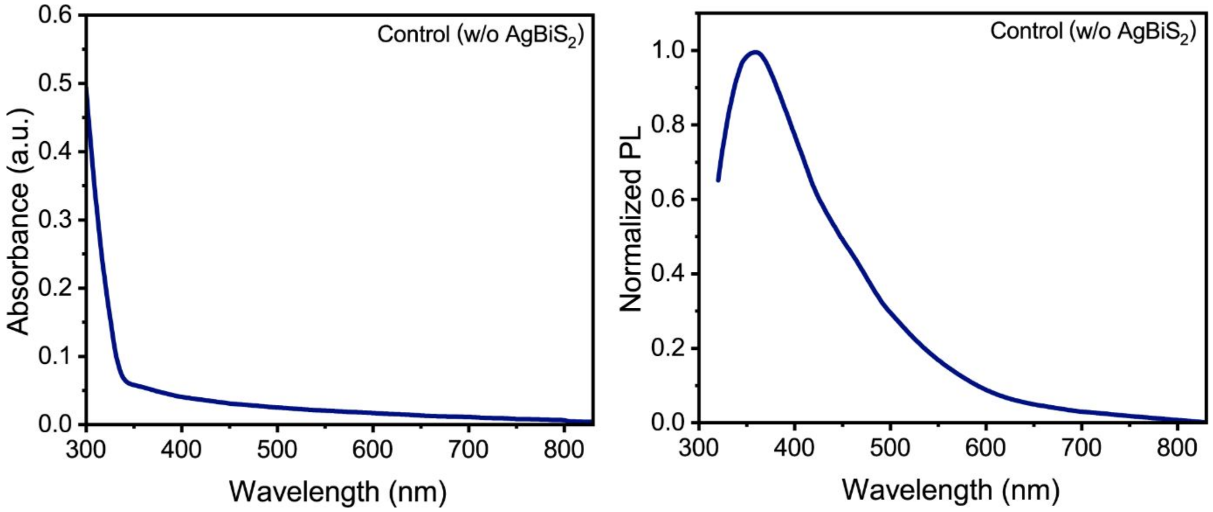

Figure S5. *Control synthesis experiment for ZnS performed without the $AgBiS_2$ core NCs. The results demonstrate the absence of (a) absorbance and (b) emission peaks characteristic of $AgBiS_2$, thereby confirming that these optical features are solely originate by the presence of the $AgBiS_2$ core in the synthesized $AgBiS_2$/ZnS NCs.*

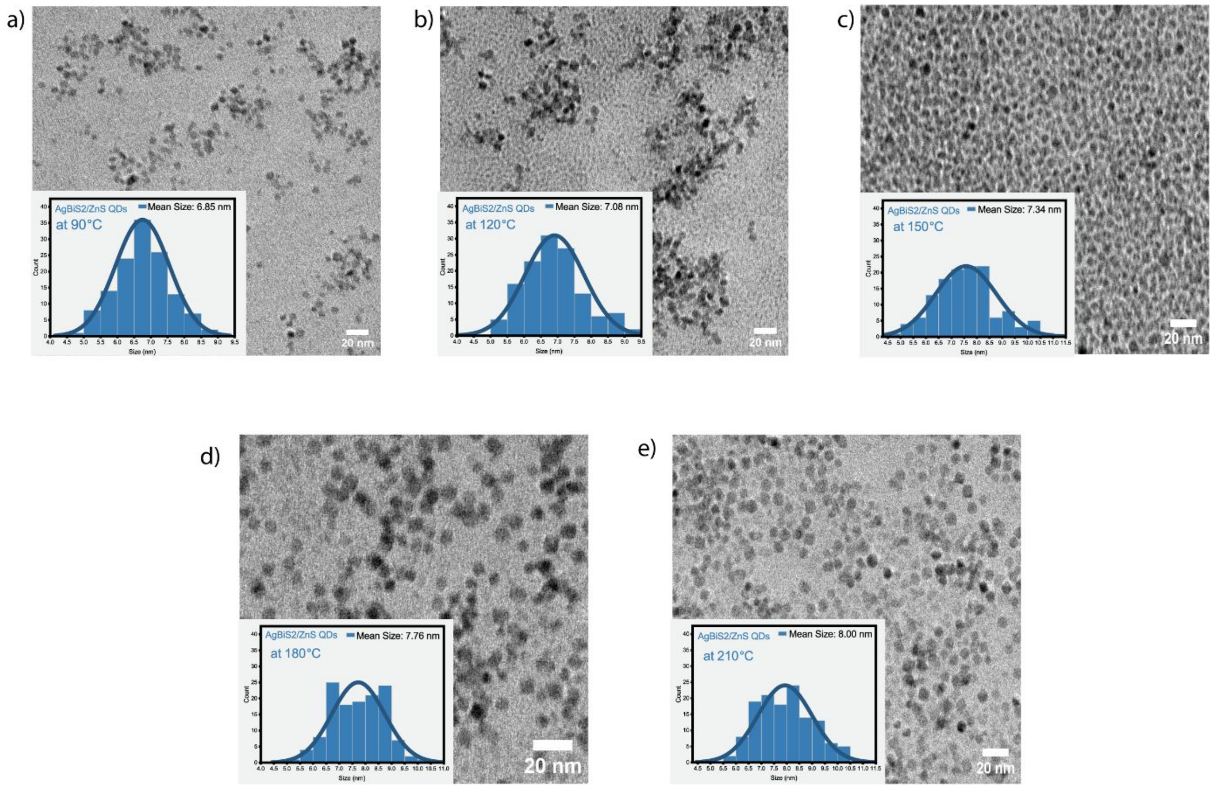

Figure S6. *TEM images of AgBiS$_2$/ZnS core/shell NCs synthesized at various temperatures, with their corresponding size distributions: (a) 90°C, (b) 120°C, (c) 150°C, (d) 180°C, and (e) 210°C.*

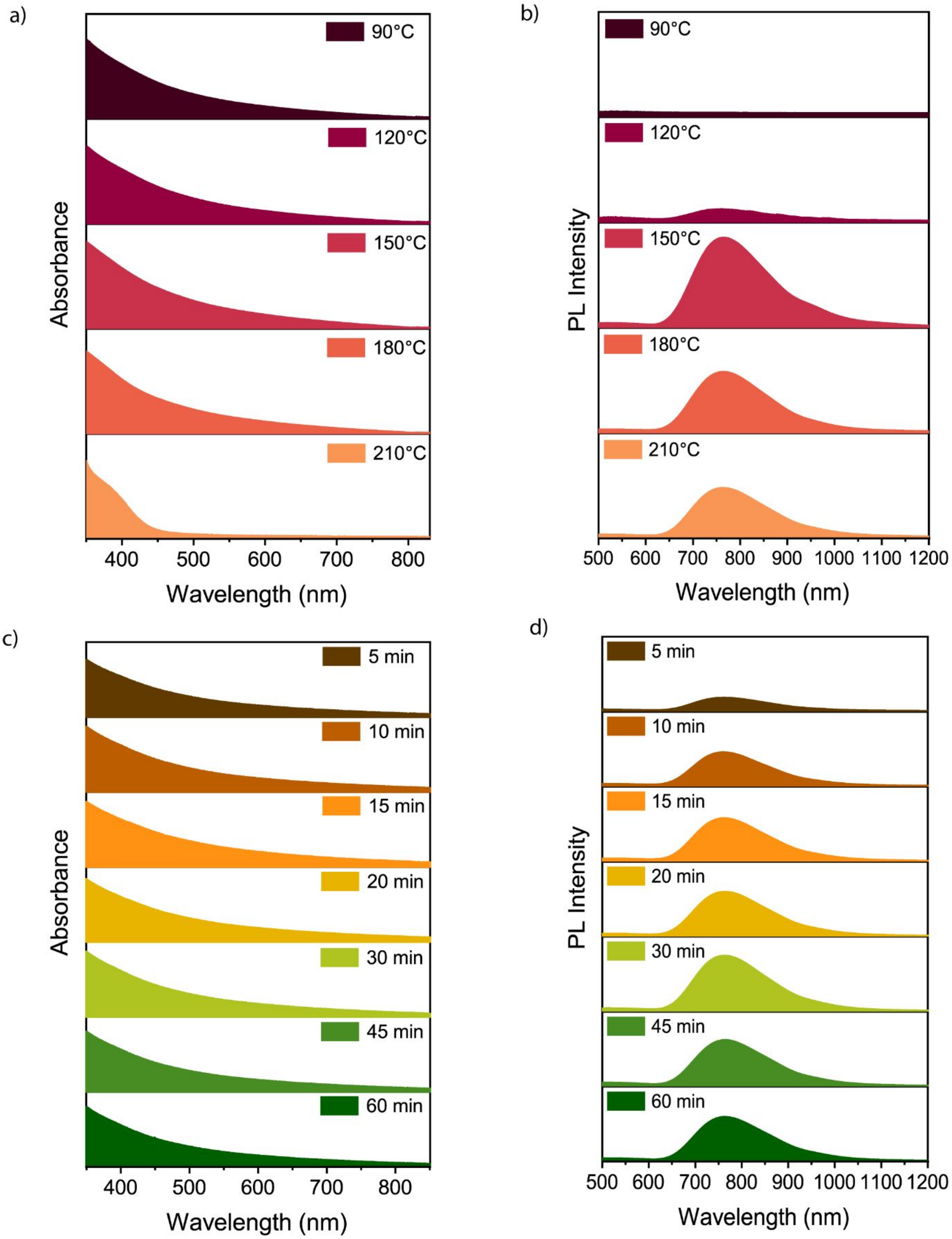

Figure S7. *UV-Vis-NIR absorption (a, c) and photoluminescence (PL) spectra (b, d) of* $AgBiS_2/ZnS$ *core/shell NCs synthesized at different temperatures (a, b) and with different reaction times (c, d), respectively.*

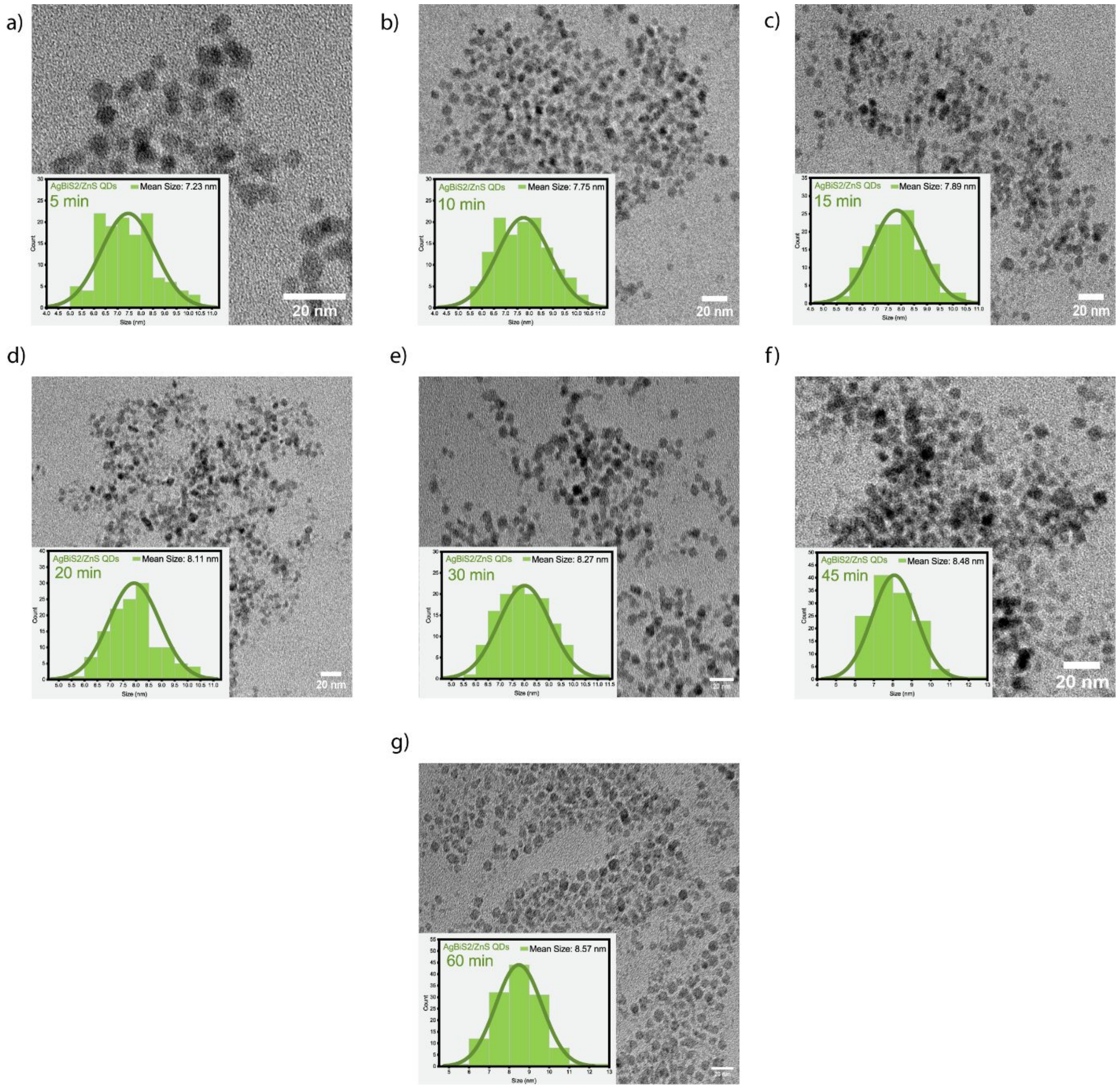

Figure S8. *TEM images of $AgBiS_2$/ZnS core/shell NCs synthesized at various reaction times, with their corresponding size distributions: (a) 5 minutes, (b) 10 minutes, (c) 15 minutes, (d) 20 minutes, (e) 30 minutes, (f) 45 minutes, and (g) 60 minutes.*

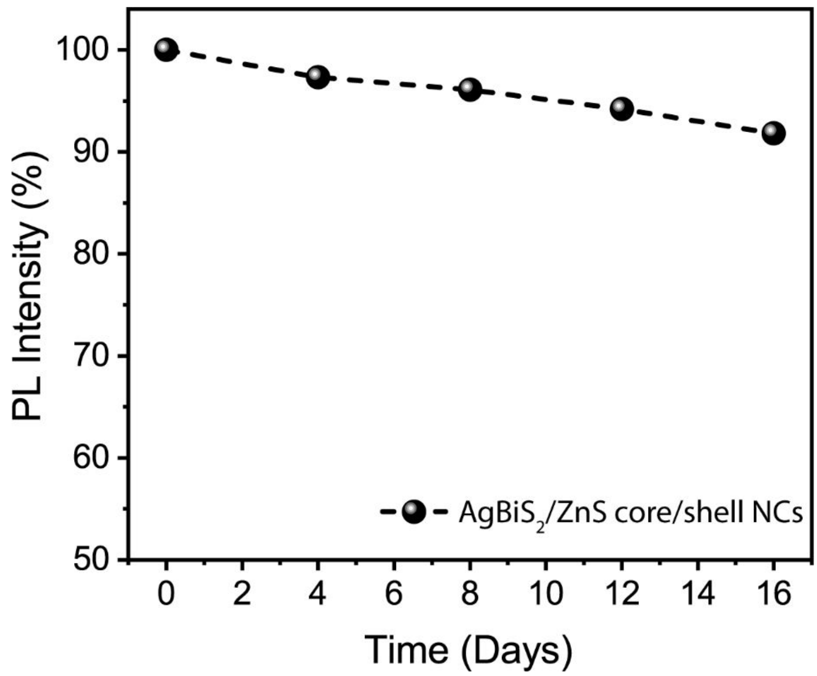

Figure S9. *Evaluation of the PL stability of $AgBiS_2$/ZnS core/shell NCs under ambient conditions.*

2. Photoluminescence quantum yield & Lifetime

2.1. Photoluminescence quantum yield (PLQY) of $AgBiS_2$/ZnS NCs

PLQY measurement of the $AgBiS_2$/ZnS NCs was performed by using an Edinburgh Instruments FLS1000 spectrofluorometer that is equipped with the Fluoracle software, which calculates the PLQY. In our measurements, the FLS1000 was equipped with an integrating sphere, which ensures accurate measurements by capturing all emitted and scattered photons, accounting for both the sample and reference spectra in a controlled environment.

As described in the Edinburgh Instruments Application Note, [1] the Fluoracle software calculates PLQY using the equation 1:

$$\mathrm{PLQY} = \frac{E_S - E_R}{S_S - S_R} \tag{1}$$

where E_S and E_R denote the integrated emission intensities of the sample and reference, while S_S and S_R are the integrated scattering intensities for the sample and reference, respectively. This approach is detailed further in the provided Edinburgh Instruments Application Note.[1] This method is fully automated within Fluoracle, ensuring consistency and minimizing user influence on calculations.

The FLS1000 setup helps mitigate the self-absorption effects by using low absorbance of the sample while maintaining it below 0.1 absorbance units in the wavelength region of interest. Additionally, the integrating sphere setup uniformly distributes light and captures emitted photons across all angles.

To support our result from the PLQY measurement, we independently reanalyzed the PLQY data using OriginLab software. Specifically, we re-plotted the data and manually calculated the PLQY by integrating the areas under the emission and scattering curves while applying the same formula utilized by the Fluoracle software (Fig. S10). This independent calculation confirmed that the PLQY values obtained were consistent with those reported by the Fluoracle software.

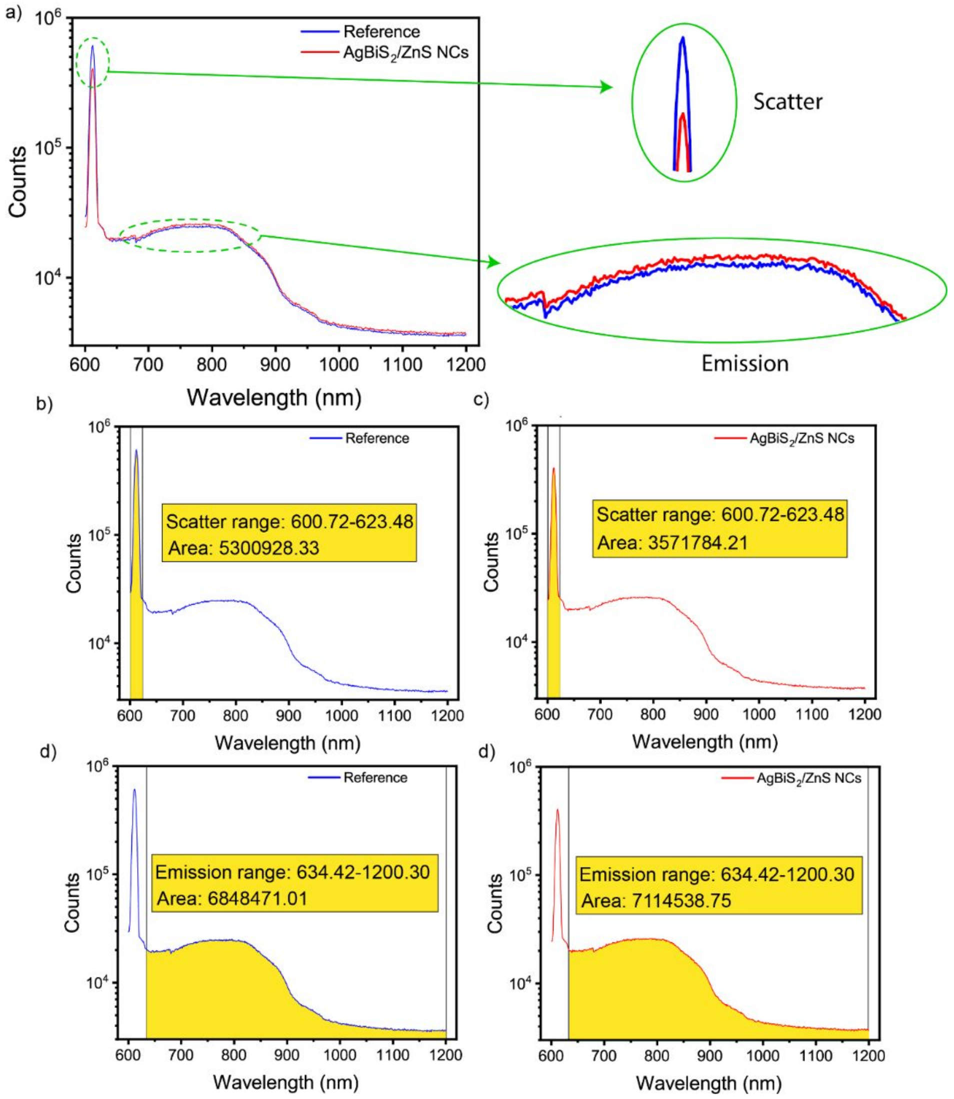

Figure S10. *(a) Scattering and emission peaks of the reference solvent (n-hexane) and* $AgBiS_2$*/ZnS NCs sample are depicted in blue and red, respectively. (b) Scattering and (d) emission peaks of the n-hexane, including their corresponding integrated areas. (c) Scattering and (e) emission peaks of the* $AgBiS_2$*/ZnS NCs sample, with their associated integrated areas. (Experimental parameters:* λ_{ex} *= 610 nm, emission range = 600–1200 nm, dwell time = 0.1 s, detector = NIR PMT.)*

2.2. PL decays and PLQY correlation

All PL decays were analyzed using a two-exponential decay model, expressed as $I(t) = I_1 \exp(-t/\tau_1) + I_2 \exp(-t/\tau_2) + I_0$.[2] This approach was adopted to account for the non-monoexponential decay characteristics observed in the NCs. The average decay lifetimes, denoted as τ_{avg}, were determined as intensity-weighted means,

$$\tau_{avg} = \frac{I_1 \tau_1^2 + I_2 \tau_2^2}{I_1 \tau_1 + I_2 \tau_2} \tag{2}$$

where lifetime components are τ_1 and τ_2, respectively. PL decays for $AgBiS_2$/ZnS core/shell NCs with varying Zn molar ratios were presented in Fig. S11, which were measured using a 594 nm long-pass filter. Average lifetimes of the NCs were calculated based on the τ_{avg} formula and are summarized in Table S2.

$$k_r = \frac{\mathrm{PLQY}}{\tau_{avg}} \tag{3}$$

$$\mathrm{PLQY} = \frac{k_r}{k_r + k_{nr}} \tag{4}$$

Radiative (k_r) and nonradiative (k_{nr}) rate constants were determined from the average PL lifetimes using equations 3 and 4.[3] The observed trends in k_r and k_{nr}, as well as the PLQY, correlate well with the PL lifetime and quantum yield measurements across different Zn molar ratios in $AgBiS_2$/ZnS NCs (Table S2). At a Zn molar ratio of 0.6, the highest PLQY of 15.3% was observed, which correspond to an increase in k_r (0.446 x10^7 s^{-1}) and a substantial decrease in k_{nr} (2.47x10^7 s^{-1}) and suggest enhanced passivation of surface defects.[3] This decrease in the nonradiative decay rate highlights the role of defect passivation in maximizing PL efficiency.[3]

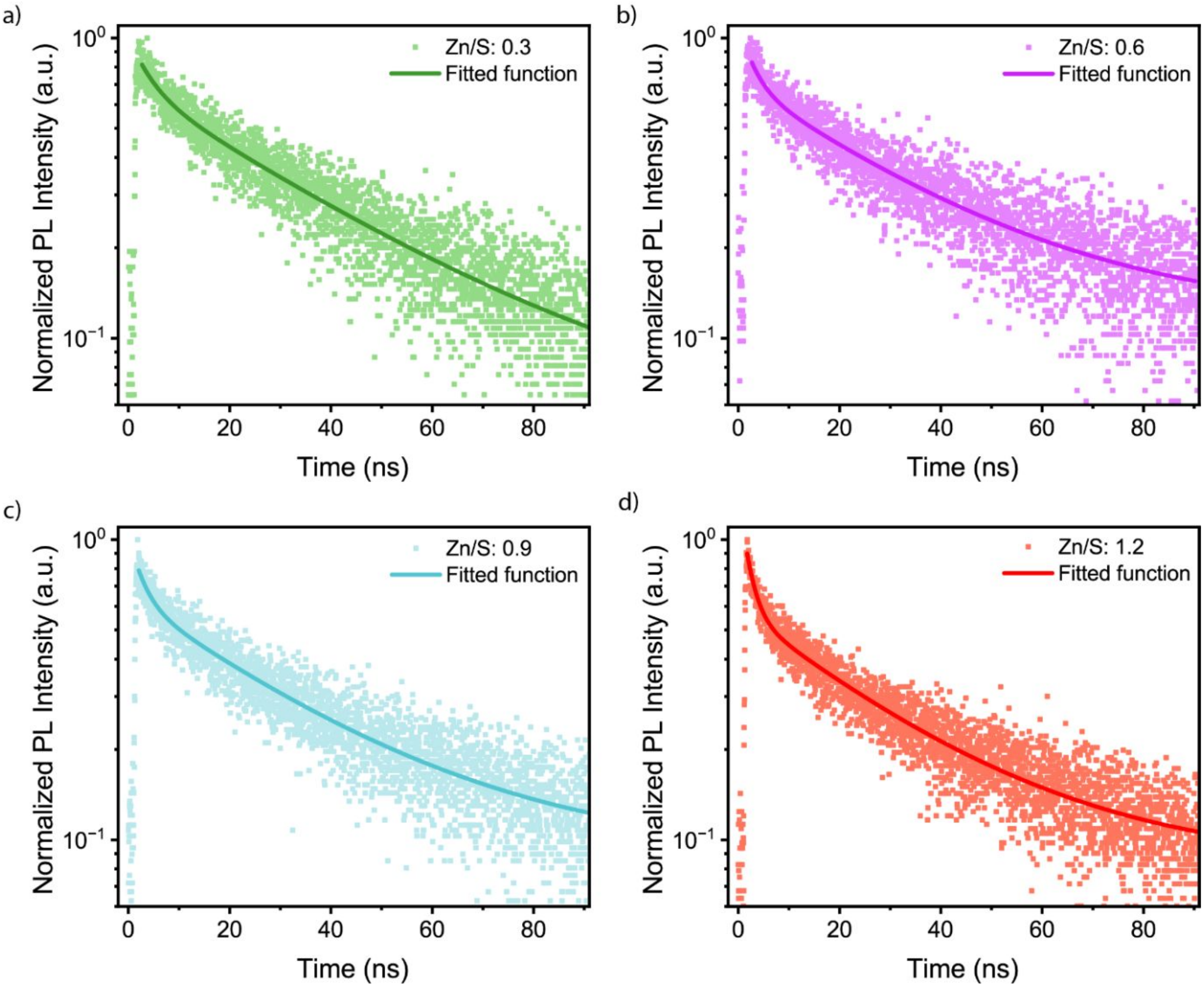

Figure S11. *PL decay curves for $AgBiS_2$/ZnS NCs at Zn/S molar ratios of (a) 0.3, (b) 0.6, (c) 0.9, and (d) 1.2. The data illustrates the changes in PL lifetimes with increasing Zn content, highlighting the impact of Zn molar ratio on radiative and nonradiative recombination processes within the NCs.*

Table S2. *PL decay results for $AgBiS_2$/ZnS NCs at different Zn molar ratios, detailing the impact of Zn content on decay dynamics.*

Sample (Zn:S)	τ_1 (ns)	I_1 (kCnts)	τ_2 (ns)	I_2 (kCnts)	τ_{avg} (ns)	k_r, 10^7 (s^{-1})	k_{nr}, 10^7 (s^{-1})	PLQY (%)
0.3	36.9	11.88	4.13	4.2	35.8	0.442	3.73	9.6
0.6	35.6	16.09	4.02	5	34.3	0.446	2.47	15.3
0.9	34.2	15.39	2.75	3.4	33.8	0.471	2.49	14.2
1.2	22.4	10.91	1.62	3.75	21.9	0.475	2.89	10.1

3. Control Experiments

3.1. XRD results of $AgBiS_2$/ZnS NCs at different reaction temperatures

Figure S12 presents the XRD analysis of $AgBiS_2$/ZnS nanocrystals synthesized at various reaction temperatures ranging from 90 °C to 210 °C. Initially, the diffraction pattern aligns closely with reference $AgBiS_2$ peaks (PDF no: 9011027), which confirms the successful synthesis of $AgBiS_2$ NCs. As the reaction temperature increases, distinct ZnS diffraction peaks (PDF no: 1100043) started to emerge and become particularly pronounced at 210 °C. This observation indicates that, beyond 180°C, ZnS not only forms a shell around the $AgBiS_2$ core but also nucleates as independent ZnS nanocrystals. Notably, even at the highest temperature of 210°C, characteristic $AgBiS_2$ diffraction peaks remain detectable, confirming the stability of the $AgBiS_2$ core throughout the shelling and temperature increase. This stability indicates that $AgBiS_2$ does not undergo degradation, even at temperatures high enough to promote the formation of ZnS nanocrystals. Moreover, the absence of any additional XRD peaks in this temperature range suggests that no byproducts were formed, supporting the conclusion that the emission observed in NIR region originates solely from the $AgBiS_2$/ZnS core/shell structure.

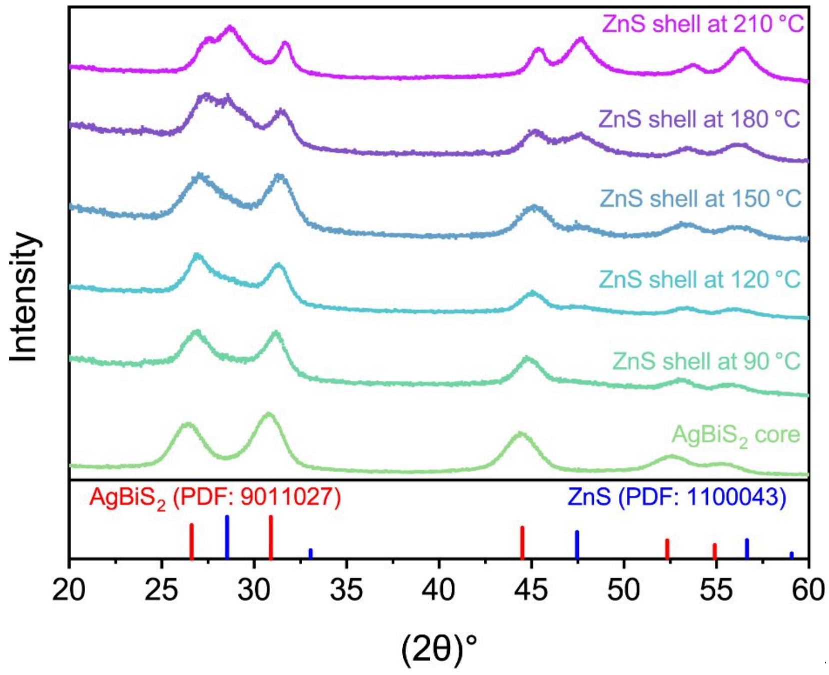

Figure S12. *XRD diffraction patterns of $AgBiS_2$/ZnS NCs at various reaction temperatures from 90 to 210℃. $AgBiS_2$ PDF card no: 9011027) (ZnS PDF card no: 1100043)*

3.2. Controlled synthesis of $AgBiS_2$/ZnS nanocrystals: Examining the impact of excluding Zn precursors

Figure S13 illustrates the optical properties of $AgBiS_2$/ZnS NCs synthesized in the absence of a Zn precursor, serving as a control to clarify the role of ZnS in emission properties. Fig. S13(a) displays the absorbance spectrum, which closely matches the absorbance characteristics observed for $AgBiS_2$ core NCs, confirming that the core structure remains unaffected in the absence of ZnS shelling. In contrast, Fig. S13(b) shows the PL spectrum, where no detectable PL emission is observed. This result demonstrates that while the PL emission originates from the $AgBiS_2$ core, it becomes observable only when the core is successfully coated with a ZnS shell. Together, these findings confirm that the emission detected in NIR region in the core/shell samples is not due to any byproducts but is linked to the $AgBiS_2$/ZnS core/shell structure.

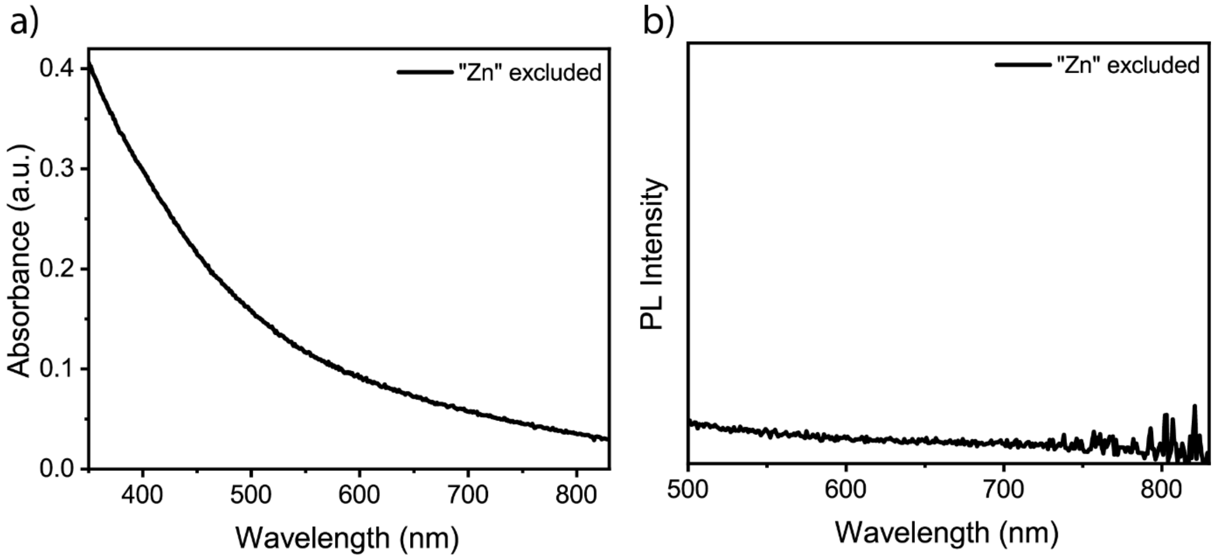

Figure S13. *(a) Absorbance and (b) PL spectra of $AgBiS_2$ NCs synthesized without the addition of a Zn precursor for shell growth as a control experiment.*

3.3. Impact of various Ag ratios on $AgBiS_2$ core NCs synthesis: Evaluation of byproduct formation in core/shell structures

Figure S14 presents XRD analysis of $AgBiS_2$ NCs synthesized at Ag precursor ratios of 2:1, 4:1, and 6:1 to illustrate the structural evolution and phase integrity of $AgBiS_2$ under varying Ag concentrations. In Fig. S14(a), the characteristic diffraction peaks of $AgBiS_2$ appeared in all samples and matched to the reference $AgBiS_2$ [PDF cards no: 9011027 and 9011025]. Notably, as the Ag content increases, a slight shift of diffraction peaks toward higher Bragg angles is observed, which indicates a reduction in the lattice parameter. This shift likely results from the partial substitution or lattice interaction of Ag^+ ions (1.15 Å) in place of larger Bi^{3+} ions (1.40 Å),[4] which induces a contraction in the crystal lattice that is consistent with ionic size effects observed in similar mixed-metal sulfide systems.[4] Fig. S14(b) displays the XRD pattern of the precipitated particles isolated from the 6:1 Ag synthesis, where diffraction peaks correspond to elemental Ag confirmed by Ag reference [PDF card 9008459] alongside with $AgBiS_2$. This result indicates that while Ag does not form Ag_2S byproducts, it precipitates independently as elemental Ag at higher Ag loadings. The inset photograph provides a visual confirmation of this separation, with Ag observed as a precipitate and $AgBiS_2$ retained in the solvent phase. These results underscore the phase purity of $AgBiS_2$ in the supernatant, which confirms that the synthesis method yields $AgBiS_2$ NCs without Ag_2S, even under conditions of elevated Ag precursor ratios.

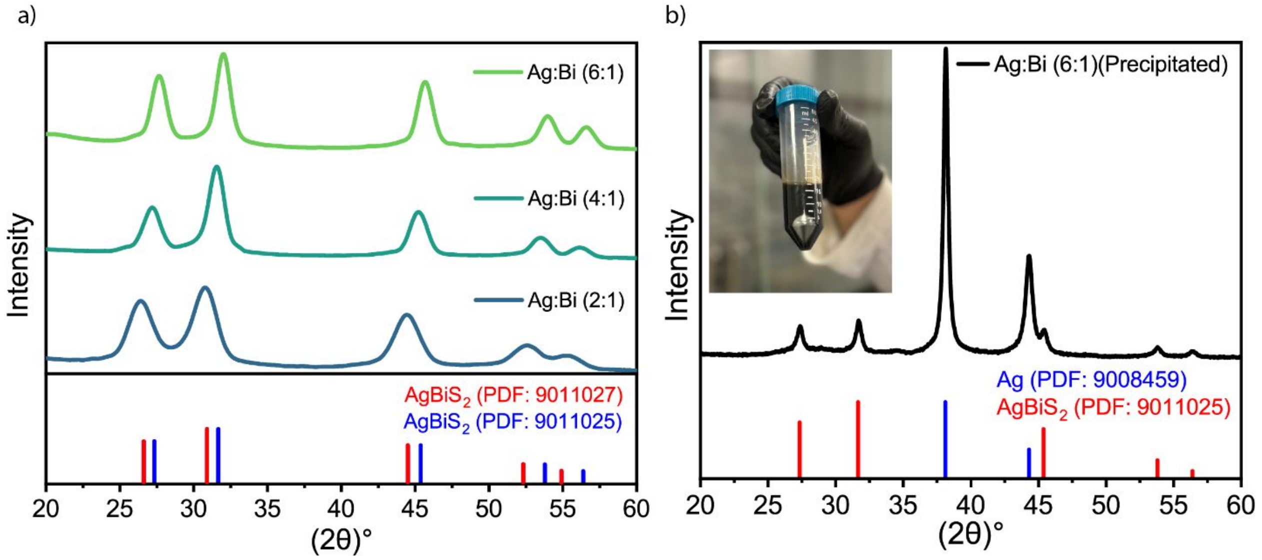

Figure S14. *(a) XRD patterns of $AgBiS_2$ nanocrystals (NCs) synthesized at various Ag ratios (2:1 to 6:1) are shown, which correspond to PDF card no. 9011027 and 9011025 for $AgBiS_2$. (b) XRD pattern of the precipitated particles from the 6:1 Ag ratio synthesis, which reveals the presence of both Ag and $AgBiS_2$ diffraction peaks (Ag PDF card no. 9008459). The inset photograph illustrates the centrifuged sample with Ag precipitates visible and $AgBiS_2$ remaining in the solvent.*

3.4. Control experiment of core/shell NCs synthesis by excluding “Bi” precursor inclusion

Figure S15 shows the XRD patterns of Ag_2S NCs before and after the ZnS shell formation. Initially, the diffraction peaks correspond to cubic Ag_2S (PDF card no. 9011414), confirming the Ag_2S phase. After the ZnS shell formation, the XRD pattern aligns solely with wurtzite ZnS (PDF card no. 2310815), with no detectable Ag_2S diffraction peaks. This change in the diffraction peaks suggests that ZnS formation dominated the reaction and lead to the degradation of the Ag_2S phase or a thick ZnS shell formation on Ag_2S core.

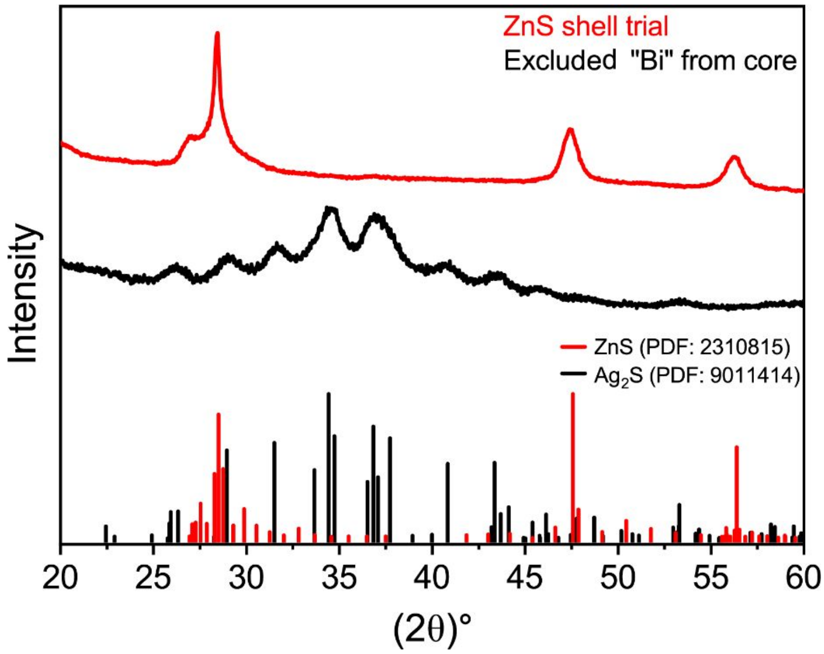

Figure S15. *XRD patterns of Ag_2S NCs before and after the application of the ZnS shell, compared with reference patterns for Ag_2S (PDF card no. 9011414) and ZnS (PDF card no. 2310815).*

3.5. Optical absorption and PL spectra of core/shell NCs synthesized by excluding "Bi" precursor inclusion

Figure S16(a) presents the absorption spectra of Ag_2S NCs before and after ZnS shell trial. The Ag_2S NCs show a typical absorption profile with broad absorbance across the visible and near-infrared regions. After the ZnS shell trial, the absorption changes with decreased intensity in the visible region and an increase toward the UV region (below 400 nm), which indicates that ZnS likely nucleated and grew independently rather than forming a shell around the Ag_2S core. Figure S16(b) shows the PL spectra of both Ag_2S NCs and the ZnS-shell tried sample under 310 nm excitation. No significant emission is observed in NIR region in either sample. The lack of emission in NIR region, along with a shift in absorption, confirms that ZnS nucleation did not result in an Ag_2S/ZnS core/shell structure, and no byproducts of Ag_2S can contribute to the observed emission in NIR by the $AgBiS_2$/ZnS system. These findings support that the emission in NIR region originates by $AgBiS_2$/ZnS NCs.

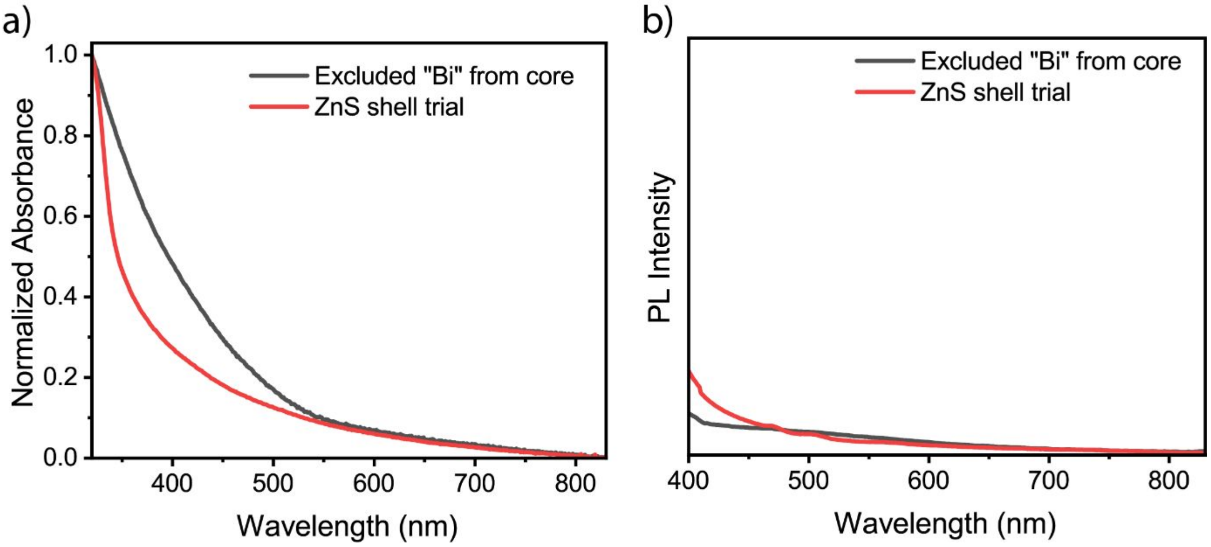

Figure S16. *(a) Normalized absorbance of Ag_2S NCs before and after the application of the ZnS shell. (b) PL spectra of Ag_2S NCs before and after ZnS shell application, with excitation at a wavelength of 310 nm.*

3.6. XRD analysis of $AgBiS_2$ core and $AgBiS_2$/ZnS core/shell NCs before and after annealing

Figure S17 presents the XRD diffraction patterns of $AgBiS_2$ NCs (Figure S17a) and $AgBiS_2$/ZnS core/shell NCs (Figure S17b) before and after annealing at 100 °C for 10 minutes. In both cases, the diffraction peaks correspond to the expected crystalline phases of $AgBiS_2$, which confirm phase stability upon annealing. The absence of additional peaks indicates that no byproducts or secondary phases were formed during the annealing process. Notably, after annealing the diffraction peaks experienced a narrowing and slight shift to larger Bragg angles. This observation aligns with the findings of Wang et al., [5] which may be attributed to enhanced crystallinity and the reduction in Ag-S bond lengths.

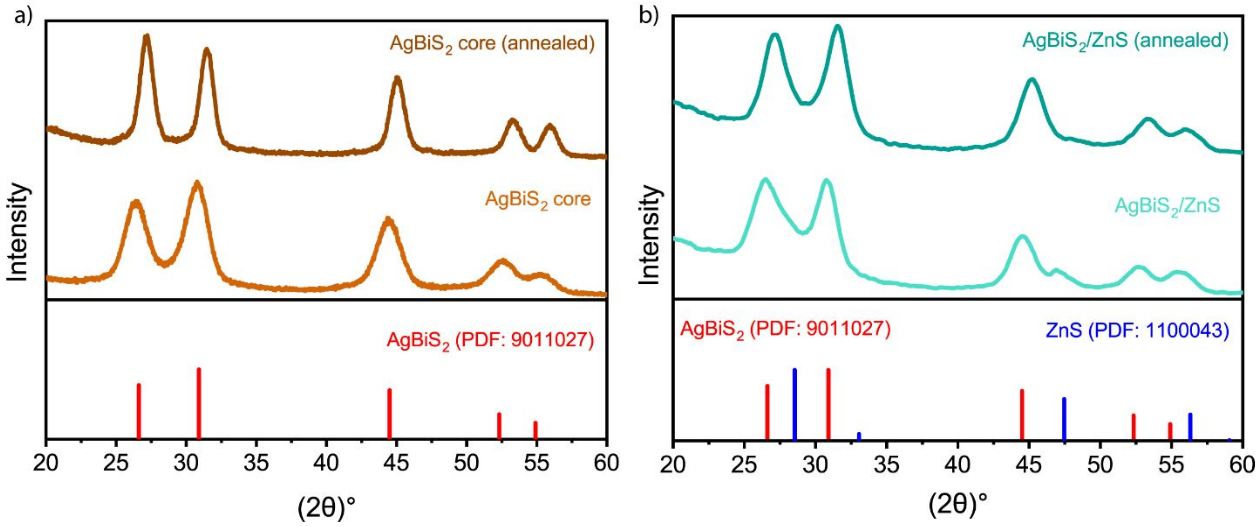

Figure S17. *XRD diffraction patterns of (a) $AgBiS_2$ NCs and (b) $AgBiS_2$/ZnS core/shell NCs, shown before and after annealing at 100°C for 10 minutes.*

References

(1) An, P.; Bailie, A. Characterisation of NIR PbS Quantum Dots Using the FLS1000 Photoluminescence Spectrometer Characterisation of NIR PbS Quantum Dots Using the FLS1000 Photoluminescence Spectrometer. **2023**, 1–3.

(2) Lunz, M.; Bradley, A. L.; Gerard, V. A.; Byrne, S. J.; Gun'Ko, Y. K.; Lesnyak, V.; Gaponik, N. Concentration Dependence of Förster Resonant Energy Transfer between Donor and Acceptor Nanocrystal Quantum Dot Layers: Effect of Donor-Donor Interactions. *Phys. Rev. B* **2011**, *83* (11), 115423.

(3) Ponomaryova, T. S.; Olomskaya, V. V.; Abalymov, A. A.; Anisimov, R. A.; Drozd, D. D.; Drozd, A. V.; Novikova, A. S.; Lomova, M. V.; Zakharevich, A. M.; Goryacheva, I. Y.; Goryacheva, O. A. Visualization of 2D and 3D Tissue Models via Size-Selected Aqueous AgInS/ZnS Quantum Dots. *ACS Appl. Mater. Interfaces* **2024**, *16* (31), 40483–40498. https://doi.org/10.1021/acsami.4c05681.

(4) Daniel, T.; Balasubramanian, V.; Grace, A. L.; Mohanraj, K. Fabrication of Ag Injection in Bi2S3 (AgBiS2) Thin Films for Photoelectrochemical Cell Applications. *Bull. Mater. Sci.* **2024**, *47* (3), 213. https://doi.org/10.1007/s12034-024-03290-5.

(5) Wang, Y.; Kavanagh, S. R.; Burgués-Ceballos, I.; Walsh, A.; Scanlon, D.; Konstantatos, G. Cation Disorder Engineering Yields AgBiS2 Nanocrystals with Enhanced Optical Absorption for Efficient Ultrathin Solar Cells. *Nat. Photonics* **2022**, *16* (3), 235–241. https://doi.org/10.1038/s41566-021-00950-4.
